# Effects of expressing a maleness gene in *Anopheles gambiae* cells using baculovirus as a gene delivery tool

**DOI:** 10.1186/s13071-026-07411-3

**Published:** 2026-05-07

**Authors:** José Héctor Gibrán Fritz García, Agata Izabela Kalita, Annika Maria Fox, Rachel Mullner, Falk Butter, M. Felicia Basilicata, Claudia Isabelle Keller Valsecchi

**Affiliations:** 1 Institute of Molecular Biology (IMB), Mainz, Germany; 2 Biozentrum, University of Basel, Basel, Switzerland; 3 Faculty of Biochemistry, Biophysics, and Biotechnology, Jagiellonian University, Kraków, Poland; 4 Institute of Molecular Virology and Cell Biology, Friedrich-Loeffler-Institute (FLI), Greifswald, Germany; 5 University Medical Center (UMC), Mainz, Germany; 6 Biomedical Center, Division of Physiological Chemistry, Faculty of Medicine, Ludwig-Maximilians-Universität (LMU), Munich, Germany

**Keywords:** *Anopheles* mosquito, Baculovirus, Sex determination, RNA helicases, Malaria

## Abstract

**Background:**

Rising global temperatures are expected to increase the spread of infectious diseases as warming allows disease-carrying mosquitoes such as *Anopheles* to survive and reproduce in new regions. To interrogate mosquito gene function rapidly and at scale, we here establish baculovirus as a gene delivery tool in *Anopheles* cells. We then use this system to express and study the dominant male sex-determining factor Yob, whose molecular function is unknown.

**Methods:**

We engineered plasmids with mosquito promoters spanning different strengths, driving genes-of-interest and selection markers, and used them to generate baculoviruses. *An. gambiae* Ag55 cells were infected over a range of virus doses and exposure durations. Infection efficiency, cell viability, and proliferation were characterized by flow cytometry. We profiled host and viral transcriptional responses by RNA-seq; assessed Yob chromatin association and localization by CUT&Tag and microscopy; and mapped protein-level changes and interactors by proteomics and immunoprecipitation.

**Results:**

We found that baculovirus infection blocks cell cycle progression and induces pronounced transcriptome changes, while *Yob* expression adds an additional male-biased signature. Yob localized to the nucleus but did not directly associate with chromatin by CUT&Tag, suggesting that it does not act as a transcription factor. *Yob* expression increased the abundance of nuclear RNA-metabolism factors, including DDX5-like helicases and CCR4-NOT-complex subunits, and Yob-HA co-immunoprecipitated with RNA-metabolism proteins.

**Conclusions:**

Together, these results demonstrate that the baculovirus system provides a versatile platform for studying mosquito gene biology in vitro. Our results imply that Yob’s mode of action is not classical DNA binding; rather, Yob is a small nuclear factor that modulates RNA-metabolism machinery, inducing a male transcriptional state.

**Graphical Abstract:**

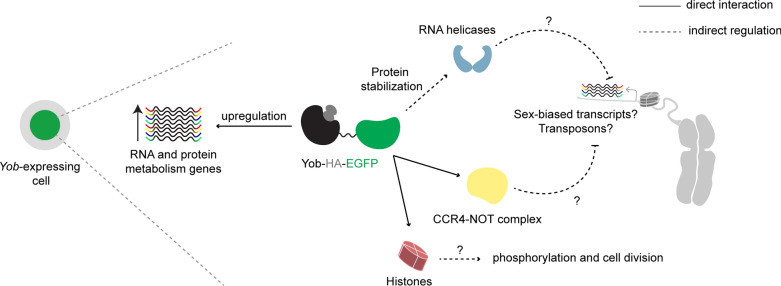

**Supplementary Information:**

The online version contains supplementary material available at 10.1186/s13071-026-07411-3.

## Background

Malaria is a deadly disease that affects hundreds of millions of people each year, primarily in sub-Saharan Africa, where roughly 95% of malaria cases and deaths occur. Global strategies aim to reduce mortality and incidence rates by 90% in 2030 [1]. Unfortunately, there is a reversal in progress, largely driven by disease-transmitting mosquitoes becoming increasingly resistant to insecticides [2]. *Anopheles* mosquitoes are the major vectors of malaria, which is transmitted when a mosquito infected with *Plasmodium* parasites feeds on human blood. Only females require blood meals to initiate egg laying and are thus capable of passing on malaria. Therefore, studying the molecular mechanisms underlying sexual dimorphism in *An. gambiae* is of high relevance to understanding malaria transmission.

Upstream regulatory factors for sex determination and sexual dimorphism evolve very rapidly, and thus, the well-studied principles governing sex determination in the model organism *Drosophila* do not apply in *Anopheles*. In *Drosophila*, sex is determined by an X chromosome counting mechanism [3]. By contrast, in *An. gambiae*, a dominant Y-linked factor only present in males, *Yob*, confers maleness [4, 5]. Ectopic expression of *Yob* by mRNA injection into embryos leads to mosquito masculinization, while its knockdown leads to partially skewed sex ratios in adults [4, 6]. In *Anopheles stephensi*, another dominant Y-linked gene, *Guy1* [7], functions to induce maleness. Although the mechanistic roles of *Yob* and *Guy1* remain unclear, they are somehow responsible for the sex-specific regulation of downstream factors like Femaleless (an RNA-binding protein inducing the female-specific splicing of *doublesex* (*dsx*) [8]), SOA (*sex chromosome activation* [5], also known as *007* [9]), the master regulator of X chromosome dosage compensation, as well as the splicing of the male-specific isoform of *dsx* [10].

Studying molecular mechanisms such as sex determination in mosquitoes presents considerable challenges. Rearing colonies requires specialized insectaries that mimic natural conditions of temperature, humidity, and light cycles. Because eggs, larvae, and pupae are aquatic, daily care is essential and pupae must be separated into emergence cages to allow adult hatching. Female mosquitoes must be fed with blood from various sources (humans, mice, rabbits, among others) in each generation to initiate egg laying, which also raises ethical concerns. Furthermore, mosquito work must comply with strict biosafety laboratory standards.

Cell culture systems have provided powerful insights into the biology of numerous organisms and could likewise be leveraged to advance mosquito research. Most existing cell lines from *An. gambiae* are female (see [11] for a complete list) and the only available male line is of muscular origin with no corresponding female line of the same cell type, limiting direct comparisons between sexes. An alternative approach could be to masculinize the existing female cells by ectopic *Yob* expression, but gene delivery in mosquito cell lines is typically low using nonviral methods [12, 13]. The baculovirus *Autographa californica* multiple nucleopolyhedrovirus (AcMNPV) expression system is widely used to produce recombinant proteins in insect and mammalian cells [14]. The system combines high infection efficiency with scalability for experiments requiring large numbers of cells (e.g., protein purification) and can accommodate one or multiple genes of virtually any size [15]. Although baculovirus has been used to deliver genes for expression in cells and mosquitoes [15], little is known about its effects on the host transcriptome, proteome, or cellular physiology. In addition, its potential to study endogenous mosquito genes, rather than reporter or viral genes (such as influenza A neuraminidase (NA3) in [15]) remains largely unexplored.

Here, we have established the baculovirus gene delivery system in *Anopheles* cells using exogenous and endogenous promoters and applied it to examine the cellular consequences of *Yob* expression in female mosquito cells. Our system provides a versatile and broadly applicable platform for studying mosquito gene function, which can also be specifically harnessed to investigate sex chromosome biology in this species.

## Methods

### Cloning of plasmids for baculovirus expression

The expression cassettes for Ag55 cells were cloned into a pFastBac Dual backbone (Thermo Fisher, 10712024) used for baculovirus generation. The pFastBac Dual backbone was modified to express (1) *mCherry* under *p10*, a viral promoter, in order to assess baculovirus efficiency and (2) a blasticidin resistance cassette under the *SV40* promoter (pFastBac-SV40p-BlastiR-p10-mCherry).

The Yob coding sequence (CDS) was obtained from genome annotation and its promoter sequence was obtained by selecting a region 1 kb upstream of the *Yob* CDS (AgamP4:Y_unplaced:28106:27107). Following the *Yob* CDS, we added a C-terminal 2× HA tag followed by a 2A cleavage site [16, 17], the *EGFP* CDS, a stop codon and a rabbit globin poly(A) signal/terminator. This fragment (YobP-YobCDS-2×HA-2A-EGFP-GlobinPolyA) was obtained as a synthetic DNA (gblock) from IDT (Integrated DNA Technologies) and was polymerase chain reaction (PCR) amplified and fused to a mouse *Pgk1* promoter contained in a plasmid reported in [18] by PCR. The fused fragment was purified and digested with *Eco*RI and *Nhe*I restriction enzymes and ligated into pFastBac-SV40p-BlastiR-p10-mCherry. The resulting plasmid, pFastBac-YobP-Yob-2×HA-EGFP-RbGlobin-PGK-BlastiR-p10-mCherry, was used for baculovirus generation; this backbone was further used to exchange the promoter upstream of *Yob*.

To replace the *Yob* promoter, the *actin 5c* promoter (Act5P) from *D. melanogaster* was amplified by PCR from pAc5.1 (Invitrogen V411020) and the *EF1a* promoter (approximately 1 kb upstream of the transcription start site of *AGAP007405*) by PCR from *An. gambiae* genomic DNA. The fragments were digested with *Eco*RI and *Fse*I and ligated into *Eco*RI-, *Fse*I-digested pFastBac-YobP-Yob-2×HA-EGFP-RbGlobin-PGK-BlastiR-p10-mCherry vector generating pFastBac-act5P-Yob-2×HA-EGFP-RbGlobin-PGK-BlastiR-p10-mCherry and pFastBac-EF1a-Yob-2×HA-EGFP-RbGlobin-PGK-BlastiR-p10-mCherry, respectively. The empty baculovirus was amplified from pFastBac-EF1a-Yob-2×HA-EGFP-RbGlobin-PGK-BlastiR-p10-mCherry using s090 and s091, digested with *Mlu*l and religated. As such, our constructs are designed to produce the protein of interest and EGFP from a bicistronic transcript via a T2A ribosome skipping event [16]. While T2A cleavage produced two separate proteins for SOA as intended (see Extended Data Fig. 5c in [5]), for Yob, the polypeptide remained fused to EGFP (Additional file 7: Fig. S3B). We speculate that the short amino acid sequence of Yob N-terminal to T2A and its positioning in the ribosome exit tunnel could presumably hamper production of two separate peptides. Plasmid sequences are provided in Additional file 1: Table S1 Plasmid sequences.

### Generation of baculovirus

pFastBac vectors with expression cassettes were transposed into the baculoviral genome using chemically competent DH10Bac cells according to the manufacturer’s protocol (Life Technologies, 10361012). Briefly, competent bacteria were thawed and incubated with 1–10 ng of bacmid, mixing by flicking several times. After incubating on ice for 30 min, heat shock at 42 °C for 45 s was performed and immediately transferred to ice afterwards for 2 min. 900 µL of SOC medium (NEB, B9020S) at room temperature was added and incubated at 37 °C for approximately 4 h with shaking (225 rpm); cells were plated in agar plates with the respective antibiotics for 48 h to allow proper distinction of white, transgene-integrated-bacmid-containing colonies from blue, non-transgene-integrated colonies. White colonies containing the transgene-integrated bacmid were used for inoculating liquid cultures with antibiotics overnight at 37 °C with shaking. 2 ml of each culture was centrifuged and bacmid was isolated from the pellet using buffers from a commercial miniprep kit (Qiagen, 27106). The pellet was resuspended in 200 μL of resuspension buffer until a homogeneous mixture was obtained. Lysis buffer (200 μL) was added and mixed by inversion, and 200 μL of neutralization buffer were added to the viscous solution and mixed by inversion. The suspension was centrifuged, and the supernatant was transferred to a clean tube. The DNA was precipitated using isopropanol (50:50), and the pellet was washed once with 70% ethanol before drying the DNA under the cell culture hood. The bacmid DNA was resuspended in 20 µL sterile water and carefully dissolved by flicking under sterile conditions/laminar flow hood.

Preparation of the baculovirus genome, transfection/P0 virus generation, and P1 virus amplification were performed as described in the Bac-to-Bac manual (Thermo Fisher Scientific), with the exception of using Cellfectin^®^ II transfection reagent (Life Technologies, 10362100) and Sf-900 III serum-free medium (Life Technologies, 12658019). Briefly, 20 µL of the bacmid DNA preparation was mixed in 200 µL of unsupplemented Grace’s Insect Cell Medium (Invitrogen, 11595-030). A second mixture of 100 µL unsupplemented Grace’s Insect Cell Medium and 6 µL of Cellfectin^®^ II transfection reagent was added to the media-diluted bacmid DNA, mixed by gently flicking the tube, and incubated at room temperature for 15–45 min.

Per well, 0.6×10^6^ SF9 cells were seeded in a 6-well plate. After 15 min, cells attached and were washed once with 2 mL of unsupplemented Grace’s Insect Cell Medium. 150 µL of the lipid:DNA complex was added to each well. After 5 h at 27 °C and 90% relative humidity, the medium was replaced with SF900 III media containing penicillin–streptomycin (Life Technologies, 15140122). Cells were further incubated for 72–96 h until mCherry signal was detected. Then, medium was collected, transferred to a tube and centrifuged at 500×*g* for 5 min. The supernatant, containing the passage 0 baculovirus particles, was transferred to a 15-mL tube and stored in the presence of 0.5% fetal bovine serum (FBS) (PAN-Biotech, P40-47). For virus amplification, 50 mL of log-phase SF9 cells at a 0.8×10^6^/mL density were infected with 1 mL of the initial passage 0 virus. Cells were incubated at 27 °C and shaken at 90 rpm for 72–96 h, while keeping the cell concentration constant through potential dilution, until a robust mCherry signal was detected for at least 75% of all cells. Again, medium was collected, transferred to a tube and centrifuged at 500×*g* for 5 min. The supernatant containing the potent passage 1 baculovirus particles was transferred to 50-mL falcon tubes and stored in the presence of 0.5% FBS at 4 °C.

### Cell culture and baculovirus infections

Ag55 cells kindly provided by Prof. M. Adang were cultured in Leibovitz L15 medium (Life Technologies, 31415029) with 10% FBS (for the baculovirus optimization, RNA-seq, CUT&Tag we used Gibco, 10270-10,6 lot: 2260092; for western blotting, IP, and microscopy we used Life Technologies, A5256701, lot: B2741829RP) and 1×penicillin–streptomycin (Life Technologies, 15140122) at 26 °C, 80% humidity. The cell line was authenticated by RNA-seq. For all flow cytometry experiments, 1 million Ag55 cells were seeded in a 12-well plate. For the infection rate experiments with different baculovirus concentrations, 24 h after seeding 50, 100, 200, 400, or 600 µL of P1 baculovirus in Sf-900 III serum-free medium was added to the cells, and the medium was changed 7 h later to fresh L15. For the infection rate experiments with different baculovirus incubation time points, 24 h after seeding, 200 µL of baculovirus in Sf-900 III serum-free medium was added to the cells and the medium was changed 2, 4, 6, 8, or 24 h later to fresh L15. For the infection rate experiments using baculovirus with different promoters, 24 h after seeding, 200 µL of baculovirus in Sf-900 III serum-free medium was added to the cells and the medium was changed 6 h later to fresh L15. For the baculovirus replication experiments, 24 h after seeding, 200 µL of baculovirus in Sf-900 III serum-free medium was added to the cells and the medium was changed 6 h later to fresh L15 and samples were collected at 24, 48, or 72 h later. For RNA sequencing experiments, 200,000 cells were seeded and the baculoviruses were added 24 h later. Cells were collected 48 h after infection. To assess if Ag55 cells faithfully produce baculovirus particles capable of infecting other cells, 1 million Ag55 or SF9 cells (positive control) seeded in a 6-well plate were infected with the “empty” or *Yob*-encoding virus (P1) at a 1:1 ratio of medium:virus, changing to a fresh virus-free aliquot 24 h later. Four days later after infection, the medium from each condition was collected and centrifuged at 500×*g* for 5 min, and the entire supernatant used to infect SF9 cells (1 million cells seeded the day before) in a 6-well plate for 24 h, before changing to a fresh aliquot of Sf-900 III serum-free medium. mCherry signal was monitored daily for 4 days.

### Cell proliferation dye staining

To test the effect of baculovirus infection on cell proliferation, cells were stained with cell proliferation dye (CPD) eFlour 450 (Life Technologies, 65-0842-85) according to the manufacturer’s instructions. Briefly, cells after infection were washed once with phosphate-buffered saline (PBS) +Ca^2+^ +Mg^2+^ and eFlour 450 was added at a final concentration of 10 µM and the cells were placed in the incubator for 10 min. The staining was stopped by adding three volumes of cold complete medium and washing once with complete medium. Finally, cells were allowed to divide for 1 additional day. For the infection rate experiments with different infection time points and with different promoter experiments, cells were stained 24 h after infection and were cultured for 1 additional day before flow cytometry. For the time point 0 h, cells were stained with CPD on the same day as flow cytometry. For viability assays, 1 mg/mL of stock propidium iodide solution (Sigma Aldrich, P4170) was added to the samples to a final concentration of 1 µg/mL.

### Cell preparation for flow cytometry and sorting

For flow cytometry analyses, cells were detached by pipetting, collected in a tube, washed once with 1× PBS with Ca^2+^ and Mg^2+^, and then resuspended in 1× PBS without Ca^2+^ and Mg^2+^. For sorting, cells were collected in a tube, washed once with 1× PBS with Ca^2+^ and Mg^2+^ and resuspended in sorting buffer (2% FBS and 1 mM ethylenediaminetetraacetic acid (EDTA) in 1× PBS without Ca^2+^ and Mg^2+^). For both setups, propidium iodide (Sigma Aldrich, P4170) was added at a final concentration of 1 µg/mL from 1 mg/mL stock solution.

### Flow cytometry and analysis

The cell suspension was analyzed with a BD LSRFortessa SORP cytometer. Side scatter (SSC) and forward scatter (FSC) were used to exclude debris, comparing FSC-area against FSC-height was used to exclude doublets and propidium iodide (561 nm; 610/20 bandpass) was used for live-dead cell exclusion. Gates for EGFP+ signal (488 nm; 530/30 bandpass) were set up on a negative control (uninfected cells) baseline fluorescence. Flowjo version 10.10 was used for analysis.

### Cell sorting

Cells were sorted with an Invitrogen Bigfoot cell sorter using a 100-µm nozzle with 30 psi in purity sort mode. SSC and forward scatter FSC were used to exclude debris, comparing FSC-area against FSC-height was used to exclude doublets, and propidium iodide (349 nm; 615/24 bandpass) was used for live-dead cell exclusion of singlets. Gates for EGFP+ signal (488 nm; 507/19 bandpass) were set up on a negative control (uninfected cells) baseline fluorescence. Before sorting, cells were filtered using a 35-µm cell strainer cap.

### Genomic DNA extraction

Infected cells (1–2 million) were collected in a tube, washed once with 1× PBS with Ca^2+^ and Mg^2+^, and resuspended in 50 µL QuickExtract™ DNA Extraction Solution (Biozym, QE09050) and incubated at 55 °C for 1 h followed by 15 min at 75 °C. Genomic DNA was either used immediately or stored at 4 °C for short-term or at −20 °C for long-term.

### RNA extraction

Media were removed from each well, and TRIzol (Life Technologies, 15596026) was added to the samples and the plate was stored at −80 °C until further processing. RNA was extracted using the Direct-zol RNA microprep kit (Zymo Research, R2062) according to the manufacturer’s protocol. Briefly, an equal volume of ethanol was added to the cells in TRIzol and the samples were transferred to the provided columns. After centrifugation (16,000×*g* for 30 s), the columns were washed and centrifuged using the same conditions with 400 µL of RNA Wash Buffer and columns were incubated with DNase I (per sample, 5 µL of enzyme in 35 µL of digestion buffer) for 15 min at room temperature. Afterwards, 400 µL of RNA PreWash Buffer was added, centrifuged, repeating the washing step once more. 700 µL of RNA Wash Buffer was added, and the columns were centrifuged for 30 s. The columns were centrifuged once more for 1 min to remove the remaining ethanol. RNA was eluted in 12 µL of nuclease-free water.

### cDNA conversion

RNA (150–300 ng) was converted to complementary DNA (cDNA) using oligo(dT) as primers and in-house made reverse transcriptase; ribonuclease inhibitor was added in the reaction (Promega, N2511).

### Quantitative PCR

For bacmid replication analysis, genomic DNA from infected cells was diluted 1:10 with nuclease-free water prior to quantitative PCR (qPCR). For gene expression changes, cDNA was diluted with nuclease-free water to a final concentration of 1.37 ng/µL. Reverse transcriptase qPCR (RT-qPCR) was performed with FastStart Universal SYBR Green Master (ROX) mix (Roche, 04913850001) in a 7 µL reaction at 300 nM final primer concentration. Cycling conditions as recommended by the manufacturer were applied. We corrected for primer efficiency using serial dilutions. Primer sequences are provided in Additional file 2: Table S2 Primer sequences.

### Protein isolation

To increase the baculovirus infection efficiency, 20 million live Ag55 cells were seeded on 10-cm plates, and the next day cells were infected with baculovirus in a 1:1 ratio of medium:baculovirus solution and incubated overnight. The morning after, the media were replaced with an aliquot of fresh medium (without baculovirus) and 2 days later cells were collected for protein extraction. The cell pellet was resuspended in lysis buffer [4-(2-hydroxyethyl)-1-piperazineethanesulfonic acid (HEPES) 50 mM, NaCl 100 mM, IGEPAL 0.05% (Sigma Aldrich, I8896), 2 mM MgCl_2_, 10% glycerol, 1× protease inhibitor cocktail (Sigma Aldrich, P8340) and 1× phosphatase inhibitor cocktail (Sigma Aldrich, P5726)], vortexed for 15 s (highest setting), and incubated for 20 min on ice, followed by centrifugation at 16,000×*g* for 10 min at 4 °C. After centrifugation, the supernatant was either used directly for western blotting or for immunoprecipitation (IP).

### Whole-cell protein extract for mass spectrometry

For proteome analysis, cells were infected as described in “Protein isolation” section, and 3 days later EGFP+ cells were sorted. Pellets were resuspended in 1× NuPAGE LDS (no dithiothreitol (DTT) added; Life Technologies, NP0007) and incubated at 80 °C for 10 min with shaking. Samples were then processed for mass spectrometry (MS).

### Immunoprecipitation

For IP, magnetic HA beads were used (Thermo Fisher Scientific, 88836). Protein concentrations were quantified using the colorimetric Bradford assay (Panreac AppliChem ITW, A6932) with a 1 mg/mL of working solution of recombinant albumin (New England BioLabs, B9200S) diluted in lysis buffer to generate a dilution curve by adding 0, 3, 6, 9, and 12 µL of the working album solution to cuvettes. To measure protein concentration in the samples, 3 µL of the extracted proteins were used. To all samples, 1 mL of the Bradford reagent was added and samples were incubated for 5 min prior to measuring. IP was performed by incubating 1 mg of total protein extract at a concentration of 1.3–1.5 mg/mL in lysis buffer (HEPES 50 mM, NaCl 100 mM, IGEPAL 0.05% (Sigma Aldrich, I8896), 2 mM MgCl_2_, 10% glycerol, 1× protease inhibitor cocktail (Sigma Aldrich, P8340) and 1× phosphatase inhibitor cocktail (Sigma Aldrich, P5726)) in a total volume of 800 µL in IP buffer (25 mM HEPES pH 7.4, 10% glycerol, 12.5 mM MgCl_2_, 0.2% Tween-20 (Sigma Aldrich, P1379), 150 mM KCl, 1× protease inhibitor cocktail and 1× phosphatase inhibitor cocktail). 5% of  the input (40 µL) was set aside prior to adding 20 µL of the beads. Samples were incubated in a cold room on a rotor for 1 h. Beads containing the HA complex were then washed three times in IP buffer, and the HA-bound proteins were retrieved by incubating the beads in 20 µL of 1× NuPAGE LDS (no DTT added; Life Technologies, NP0007) at 42 °C for 10 min with shaking. The denatured proteins were retrieved by binding the beads to an ice-cold magnetic rack and transferring the supernatant containing the proteins to a clean tube. The process was repeated once more by resuspending the beads in 10 µL of LDS (no DTT added), and supernatant was collected in the same tube as before. DTT was added to a final concentration of 50 mM, and samples were incubated at 70 °C with shaking for 10 min.

### Western blotting

Protein extracts were mixed with 4× NuPAGE LDS (Life Technologies, NP0007) supplemented with 50 mM DTT and incubated at 70 °C for 10 min. Approximately 30 µg of proteins were then separated by a 10% self-casted gel in 1× 3-(*N*-morpholino)propanesulfonic acid (MOPS) buffer. Gels were transferred to a 0.45 µm polyvinylidene fluoride (PVDF) membrane in Tris–glycine transfer buffer with 10% methanol (2 h at 0.4 A). Membranes were blocked for 30 min in 3% bovine serum albumin (BSA) (PAN Biotech, P06-1391500) in PBS-0.1% Tween before incubation with primary antibodies in 5% milk in PBS-0.1% Tween overnight at 4 °C. Secondary horseradish peroxidase (HRP)-coupled antibodies were incubated for 1 h. Blots were developed using SuperSignal West Pico PLUS Chemiluminescent Substrate (Thermo Scientific, 34577) or SuperSignal™ West Atto Ultimate Sensitivity Substrate (Thermo Scientific, A38554) and imaged on a ChemiDoc XRS + (Bio-Rad). Primary and secondary antibodies used are provided in Additional file 3: Table S3 Antibodies.

### Immunofluorescence staining

Infected cells growing on uncoated coverslips were washed once with 1× PBS without Ca^2+^ and Mg^2+^ and fixed with 4% methanol-free formaldehyde (Thermo Scientific, 28906) in 1× PBS without Ca^2+^ and Mg^2+^ at room temperature for 15 min, followed by two washes of 5 min each in 1× PBS without Ca^2+^ and Mg^2+^. Samples were either processed immediately or stored at 4 °C in 1 × PBS without Ca^2+^ and Mg^2+^ until further use.

Fixed cells were permeabilized for 2 min 30 s in 0.25% Triton X-100 (Thermo Fisher, BP151-100) in 1× PBS without Ca^2+^ and Mg^2+^. Coverslips were then washed three times with 1× PBS without Ca^2+^ and Mg^2+^ and cells were counterstained with 4′,6-diamidino-2-phenylindole (DAPI) (Fisher Scientific GmbH, 10184322, 1:2000) for 10 min at room temperature, rinse once with rinsed 1× PBS without Ca^2+^ and Mg^2+^, then rinsed once with milliQ water and finally rinsed again with 1× PBS without Ca^2+^ and Mg^2+^. Coverslips were mounted on slides with VECTASHIELD Vibrance^®^ Antifade Mounting Medium (Vector Laboratories, H-1700-2). Imaging was performed on the same day.

### Microscopy and image processing

Images were acquired by taking *Z*-planes using a fluorescence spinning disc confocal microscope, VisiScope 5 Elements (Visitron Systems), which is based on a Ti-2E (Nikon) stand and equipped with a spinning disc unit (CSU-W1, 50 μm pinhole; Yokogawa). The setup was controlled using VisiView 5.0 software, and images were acquired with a 100×/1.49 NA oil-immersion objective and a sCMOS camera (BSI; Photometrics). Three-dimensional (3D) stacks of images were recorded for each sample. Raw.companion.ome files were processed using Fiji (ImageJ2, version 2.16) and converted to .tif files. The intensity of all channels was equally adjusted for all images per replicate, and the resulting .tif merged files containing maximum intensity projections were produced from *Z*-stacks and further used to quantify mean fluorescence. Quantification was performed by placing equally sized squares over the nucleus (based on DAPI staining) and cytoplasm of the same cell. This was repeated for all EGFP+ cells in each field of view and across all images. Using Fiji’s ROI Manager Multi-Measure function, mean intensity values were extracted for all channels and regions. Nuclear enrichment per cell was calculated as the ratio of mean nuclear to mean cytoplasmic intensity. Finally, the average ratio for all cells in each field of view was computed, and each data point in the plots represents a single acquired image.

### CUT&Tag library generation and sequencing

CUT&Tag was performed as previously described [19]. In total, 0.4×10^6^ cells were used for each reaction. Cells were freshly collected and processed with the native protocol. We used pA–Tn5 prepared by the IMB Protein Production Core Facility and 15 PCR cycles in the library amplification step. Pooled samples were sequenced on NextSeq 500 High Output, PE for 2×75 cycles plus 2×8 cycles for the dual index read. Primary and secondary antibodies used are provided in Additional file 3: Table S3 Antibodies.

### CUT&Tag data processing and analysis

Reads were trimmed using cutadapt (version 4.0) to remove Illumina adapter sequences and subsequently mapped to the reference genome with bowtie2 (version 2.4.5). We then called peaks using macs2 (version 2.1.2) with the corresponding IgG control samples used to construct a graylist for peak filtering. This provided consensus peaks for downstream analysis with DiffBind (version 3.6.1) to identify sites that were significantly (false discovery rate (FDR) < 0.05) differentially bound between samples. Background bins instead of library size were used for DiffBind normalization. Coverage signal tracks (bigWigs) and downstream visualization of differentially bound peaks (for example, metaplots) were generated using deepTools (version 3.5.1). Downstream analysis and statistical tests were performed using R studio. Coverage tracks were visualized in IGV (version 2.19.4).

### RNA sequencing analysis and data visualization

The reads were mapped to the merged Ensembl AgamP4 genome and baculovirus genome (L22858.1) using the Ensembl AgamP4 annotation (release 48) together with long noncoding RNA (lncRNA) annotation [20] with STAR (version 2.7.3a) using the following parameters: outFilterMismatchNoverLmax 0.04 and outFilterMismatchNmax 999. Only uniquely mapped reads were used for downstream analysis. Coverage signal tracks (bigWigs) of primary alignments were generated using deepTools (version 3.1.0). Primary alignments were assigned to features using subread (version 1.6.5) with the AgamP4 annotation (release 48) combined with lncRNA annotation [20] and annotation of the baculovirus genome as a reference. Differential expression analysis was performed using DESeq2 (version 1.26.0), and genes with FDR < 0.05 were considered as differentially expressed. For gene ontology analyses, the differentially expressed genes were imported to VectorBase and analyzed using the GO term tool, selecting Biological Processes. Terms meeting *P*_adj_ < 0.05 (Benjamini–Hochberg correction) were considered significantly enriched and further used for plotting. To identify the gene ontology analyses from the *Anopheles* X chromosome, all X-linked genes were extracted with VectorBase and further imported to the GO term tool as specified before. The list of terms was then exported.

### Sample preparation for MS

For MS, samples were processed via in-gel digestion as described previously [21]. Denatured proteins were loaded onto a 4–12% NuPAGE Bis-/Tris Gel (Thermo Scientific) and run at 180 V for 8 min in 1× MES sodium dodecyl sulfate (SDS) running buffer (Thermo Scientific). Proteins in the gel were fixed with MS fixation buffer (7% acetic acid and 40% methanol) for 10 min and subsequently stained with Coomassie Blue [10% acetic acid, 45% ethanol, 0.25% w/v brilliant blue (Roth)] for 5 min. The gels were destained overnight in distilled water. Each sample of the gel was cut into 1×1 mm^2^ squares, added to a 96-well filter plate (Millipore Sigma) and further destained through repeated rounds of 50% ethanol and ammonium bicarbonate at final concentration of 25 mM (ABC, pH 8.0) at 300 rpm and 37 °C for 15 min. The gel pieces were dehydrated with three rounds of 100% acetonitrile with shaking at 300 rpm for 10 min at 25 °C. The disulfide bridges were reduced with DTT at final concentration of 10 mM (Sigma) in 50 mM ABC at 56 °C for 1 h. The reduced proteins were alkylated with iodoacetamide (IAA) at final concentration of 50 mM (Sigma) in 50 mM ABC at 25 °C for 45 min in the dark. The gel pieces were washed with 50 mM ABC (pH 8.0) at 300 rpm and 25 °C for 20 min. The gel pieces were again dehydrated with three rounds of 100% acetonitrile with shaking at 300 rpm for 10 min at 25 °C. The gel pieces and filter plate were dried at 85 °C for approximately 20 min. Proteins in the gel pieces were digested overnight at 37 °C with 1 µg MS-grade trypsin (Serva) in 50 mM ABC, pH 8.0. Digested peptides were eluted from the gel pieces through two rounds of extraction buffer (30% acetonitrile in 50 mM ABC), shaken at 300 rpm at 25 °C for 15 min, and then collected in a 96-well deep plate (Eppendorf) through centrifugation at 500×*g* for 2 min. Acetonitrile was added to the gel pieces three times, with shaking at 300 rpm for 10 min at 25 °C, followed by centrifugation at 500 × *g* for 2 min. Eluted peptides were transferred to 1.5-mL tubes (Eppendorf) and placed in a concentrator (Eppendorf) for 2 h to evaporate the acetonitrile until the remaining volume was less than 200 µL. Peptides were desalted with StageTips as described previously [22]. StageTips were prepared by stacking two layers of Empore C18 material (3 M) and activating with 50 µL of methanol. The StageTips were equilibrated with 50 µL of Buffer B (0.1% formic acid, 80% acetonitrile) and washed with 50 µL of Buffer A (0.1% formic acid). Peptides were added to prepared StageTips and centrifuged at 500×*g* for 7 min. Peptides on the StageTips were washed with 50 µL of Buffer A. To elute peptides, 30 µL of Buffer B was added to the StageTips, which were then centrifuged at 500×*g* for 2 min and collected in a 24-well MS loading plate (Thermo Scientific). The MS loading plate was placed in a concentrator to evaporate the acetonitrile until the remaining volume was 7 µL. Seven microliters of Buffer A (0.1% formic acid) were added to the samples to achieve a final volume of 14 µL. Pulldown samples were left undiluted and proteome samples were diluted to 200 ng/µl in Buffer A.

### MS data acquisition and analysis

Liquid chromatography–tandem mass spectrometry was performed with a NanoElute coupled to a timsTOF-HT (Bruker), and MS data were acquired via Compass HyStar and timsControl. Sample (2 µL) was injected and separated on an Aurora Series 3 column [25 cm length, 75 µm inner diameter, 1.7 µm C18 (IonOpticks)] in one column separation mode with a flow rate of 300 nL/min over a 40 min gradient for pulldown samples and a 100 min gradient for proteome samples containing a mixture of Buffer A (0.1% formic acid in water) and Buffer B (0.1% formic acid in acetonitrile) described as follows: 2 min 2% Buffer B, 28 min 4% Buffer B, 6 min 17% Buffer B, 1 min 32% Buffer B, and 3 min 95% Buffer for the 40 min gradient and 2 min 2% Buffer B, 65 min 4% Buffer B, 21 min 17% Buffer B, 4 min 27% Buffer B, 5 min 95% Buffer B, and 3 min 95% Buffer B for the 100 min gradient. The column temperature was maintained at 50 °C via a column toaster (Bruker). The separated peptides were ionized with a CaptiveSpray ion source at a capillary voltage of 1600 V, a dry temp of 180 °C, and 3.0 L/min dry gas. MS data were acquired in positive ion and data-dependent acquisition–parallel accumulation serial fragmentation (DDA-PASEF) mode with ten PASEF MS/MS scans covering a mass range of 100–1700 *m/z*.

The raw MS data were analyzed via MaxQuant version 2.4.2.0 with the default settings [23]. Under group-specific parameters, trapped ion mobility spectrometry (TIMS)-DDA was selected. Match between runs was activated for pulldown samples. Proteins were quantified via the LFQ algorithm (without FastLFQ) with a minimum ratio count of two unique + razor peptides for pulldown samples and two unique peptides for proteome samples. Proteins were identified via database matching with the Anopheles gambiae.AgamP4 database (15,125 entries, ENSEMBL), with an FDR of 0.01. Contaminants, protein groups identified only by site, reverse database hits, and proteins with fewer than two peptides were removed. Missing values were imputed. Statistical analysis and figures were performed with R.

### AlphaFold modeling

To obtain protein secondary structures, the amino acid sequences of the untagged wild-type Yob and tagged Yob-HA-EGFP were run independently using AlphaFold [24, 25] on Tamarind Bio (https://www.tamarind.bio/), and the confidence scores (pIDDT) were automatically generated by the software. The provided .pdb files were aligned using the Research Collaboratory for Structural Bioinformatics (RCSB) Protein Data Bank alignment tool [26].

### Statistics and reproducibility

Flow cytometry data for baculovirus optimization were obtained from at least *n*=2 independent biological replicates; at least *n*=2 biological replicates for qPCR data to assess baculovirus replication; *n*=3 independent biological replicates to assess re-infection capability of particles produced by Ag55 cells; *n*=2 independent biological replicates for CUT&Tag with the HA antibody and one biological replicate for H3K27me3; *n*=4 independent biological replicates for RNA-seq and MS experiments; at least *n*=2 independent biological replicates for protein extractions; *n*=2 independent biological replicates for quantification of EGFP mean intensity fluorescence; *n*=4 independent baculovirus infection replicates together with three male and three female pupae for assessing *dsx* splicing upon *Yob* expression compared with wild-type animals (RT-qPCR). Statistical analyses were performed in R; information on respective statistical tests is provided in figure legends.

## Results

### Baculovirus expression system for *Anopheles* cell expression

**Fig. 1:**
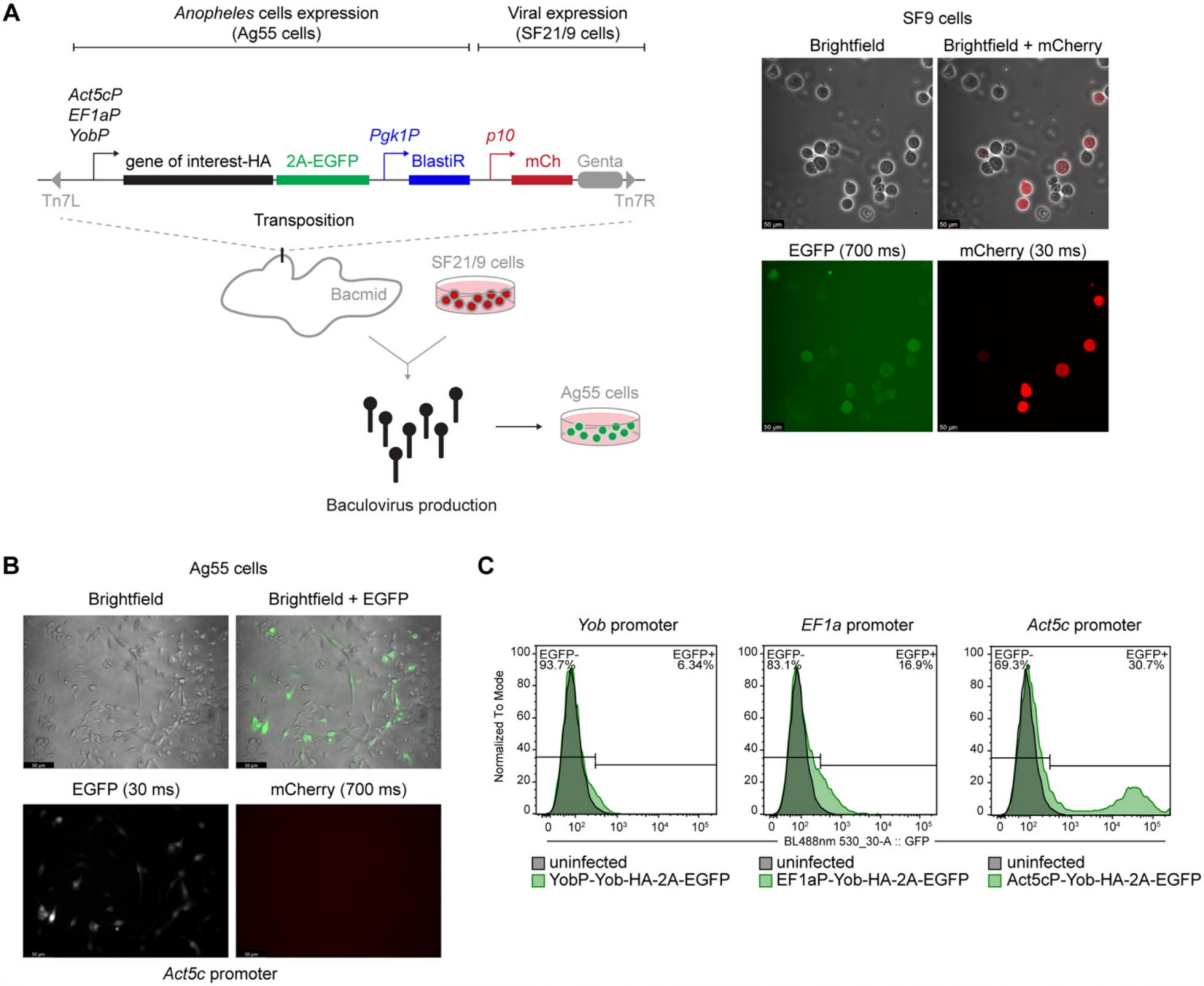
Establishment of baculovirus as a gene delivery tool in Ag55 cells. **A** Left: engineered version of the baculovirus genome encoding an HA-tagged gene/protein of interest under different promoters. A gene of interest is put upstream to the *EGFP* coding sequence separated by a 2A peptide. After transposition of the insert, the bacmid is transfected to SF21/9 cells to produce live baculoviruses. Right: Microscopy image of SF9 cells infected with *Act5c-Yob-2A-EGFP*, *p10-mCh* baculovirus. Exposure of each fluorescent channel is provided. Scale bar: 50 µm. **B** Representative image of detection of infected EGFP+ Ag55 cells. Exposure of each fluorescent channel is provided. Scale bar: 50 µm. **C** Representative flow cytometry histogram of infected Ag55 cells with *Yob*-expressing baculovirus under different promoters. *Act5cP, Actin 5c* promoter; *BlastiR,* blasticidin resistance gene; *EF1aP, EF1a* promoter; *Genta,* gentamycin resistance gene; *mCh, mCherry;* *ms,* milliseconds; *Pgk1P, Pgk1* promoter; *YobP, Yob* promoter

**Fig. 2:**
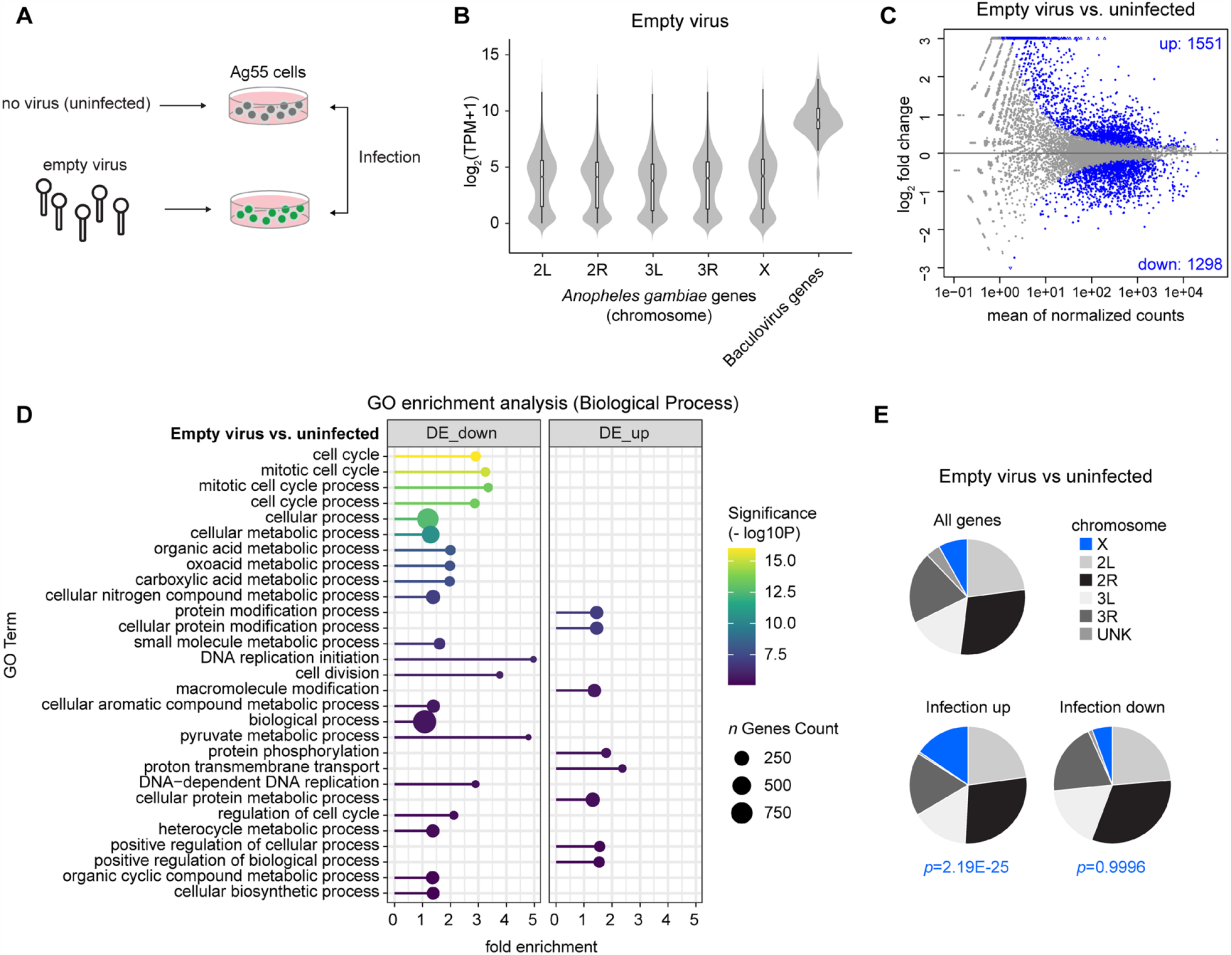
Gene expression changes upon baculovirus infection. **A** Scheme of the experimental setup. **B** Violin plots of log_2_(FC) values of Ag55 cells infected with an “empty” virus. The center line of the boxplot indicates the median. **C** MA plots from RNA-seq showing normalized read counts versus log_2_(FC) comparing cells infected with an “empty virus” against uninfected cells. Blue dots indicate differentially expressed genes (FDR<0.05). **D** Gene Ontology (Biological Process) analysis of the differentially expressed genes from RNA-seq of Ag55 cells infected with an “empty virus” versus uninfected cells. Upregulated and downregulated gene sets (FDR<0.05) were analyzed independently, and GO terms meeting *p*_*adj*_ < 0.05 (Benjamini–Hochberg correction) were considered to be significantly enriched. The lollipop plot shows the fold enrichment of genes in the various classes, with the point size indicative of the gene count and color indicative of the significance. The analyses were conducted with the GO Term tool on VectorBase. **E** Pie chart of the proportion of differentially expressed genes belonging to each mosquito chromosome comparing cells infected with an “empty virus” against uninfected cells. *P* values were obtained using a one-sided Fisher’s test for overrepresentation. *DE,* differentially expressed; *TPM,* transcripts per million; *UNK,* unknown

To establish baculovirus as a gene delivery system in *An. gambiae*, we used the female Ag55 cell line—derived from neonate first-instar larvae [27]—as an experimental model. We generated plasmids containing three elements: (1) a mosquito cell expression cassette driven by either the weak *An. gambiae YobP*, medium *An. gambiae EF1aP* or strong *D. melanogaster Act5cP* (Fig. 1A; left panel) which co-expresses an HA-tagged gene of interest and *EGFP* from the same mRNA via a T2A ribosome skipping sequence [16, 17] (Fig. 1B); (2) a blasticidin resistance gene driven by the mouse *Pgk1* promoter, which is active across insect cells (including *Anopheles*, see below) and allows for both selection and relative virus quantification [28] (Fig. 1A; left panel); and (3) an *mCherry* fluorescent marker driven by the AcMNPV *p10* promoter for monitoring baculovirus production in SF9/21 cells (Fig. 1A; right panel). By Tn7 transposition, the plasmids are integrated into a bacmid genome that can then in turn be used for generation of baculovirus by transfection into lepidopteran SF9 cells. The baculoviruses are harvested from the tissue culture supernatant and can then be used to infect mosquito Ag55 cells. We used this strategy to express *Yob* (Fig. 1C) and *SOA* (*SOA* is not described here, see [5]), which encode two proteins which are normally not present in female Ag55 cells. For comparison we generated “empty” negative virus controls only expressing EGFP (referred to as “empty virus”) from the aforementioned promoters to assess the effects of viral infection versus the expression of the transgene.

### Establishment of baculovirus-mediated gene expression in *Anopheles* cells

We first evaluated promoter strength by measuring the green fluorescence encoded in the constructs. As expected, EGFP quantification by flow cytometry showed that medium to strong promoters (*EF1aP* and *Act5cP*, respectively) produce readily detectable EGFP+ cells (proportion and intensity) compared to the weak *YobP* promoter that had fluorescence levels close to background (Fig. 1C). The fluorescence intensities driven by *EF1aP* and *Act5cP* were clearly distinguishable from the negative control, also allowing efficient fluorescence-activated cell sorting (FACS) (see below). We next wanted to understand how different conditions affect infection efficiency, while evaluating cell survival and cell proliferation. For this, we focused on the *Act5cP*-driven *Yob*-*HA* construct. Ag55 cells were infected, and the medium was refreshed with a virus-free aliquot at different time points (Additional file 4: Fig. S1A). Prolonged incubation with the baculovirus increased the proportion of *EGFP*-expressing cells, reaching a maximum at 8 h (Additional file 4: Fig. S1B). Cell viability, assessed by propidium iodide staining, moderately decreased from around 95% to around 90% when the cells were incubated for 8 h or longer (Additional file 4: Fig. S1B). Using 6 h as the incubation time that resulted in a high efficiency without compromising viability, we further measured how increasing amounts of baculovirus impact infection rate. The proportion of EGFP+ cells increased with a higher viral load (Additional file 4: Fig. S1C). These results suggest that the amount of baculovirus added, rather than the incubation time, is a key determinant of efficiency.

Baculovirus can replicate in lepidopteran SF9/SF21 cells (e.g., moths and butterflies), reaching a full reproductive cycle from DNA replication to virion production, while this is not the case in mammalian cells [29, 30]. In *Aedes* and *Drosophila* cells, the baculovirus is able to replicate but typically fails to complete the full cycle [15, 30]. Different from SF9 cells (Fig. 1A), Ag55 showed no *mCherry* expression (Fig. 1B), indicating that the late *p10* promoter was not functional in *Anopheles*. We infected the cells with the “empty virus” or *Yob*-expressing baculovirus for 6 h, changing to fresh medium aliquot without virus afterwards. We then quantified the viral DNA levels relative to endogenous *An. gambiae* genes by qPCR at 24, 48, and 72 h post-infection. DNA levels of viral genes increased over time in cells treated with the “empty virus,” suggesting that the virus can replicate (Additional file 4: Fig. S1D). Cells infected with an *SOA*-expressing baculovirus (generated in [5]) behaved similarly. Interestingly, the opposite was observed when cells were treated with the *Yob*-expressing baculovirus, where the viral genome decreased over time compared to the *An. gambiae* genes. This implies that baculovirus replication in Ag55 could be directly affected by the protein of interest expressed, or that cells expressing it would be selected against. With regards to Yob, we interpret these results to suggest that triggering “maleness” induces a detrimental cellular response specifically in infected female cells, and that uninfected cells are concurrently subjected to positive selection.

Since we observed that Baculovirus can enter the Ag55 cells and replicate at least under some circumstances, we also wanted to test whether infection of the replication-competent “empty” or *Yob*-expressing virus led to the faithful production of virions particles. For this, we used the supernatant of infected Ag55 (4 days) or SF9 cells (positive control, as they can produce viral particles) to reinfect SF9 cells. We monitored *mCherry* expression daily to assess successful reinfection in the lepidopteran host cells. As expected, SF9 cells infected with the supernatant of previously infected SF9 cells with both viruses led to mCherry signal as early as 2 days. mCherry signal was also seen in SF9 cells infected with the supernatant of previously infected Ag55 cells, indicating that indeed viral particles can be produced in *An. gambiae* cells (Additional file 4: Fig. S1E).

### Baculovirus infection decreases cell proliferation in Ag55 cells

Baculovirus infection blocks the cell cycle, preventing entry into mitosis [31], and consistent with this, previous work reported a cell division delay in an *Aedes* mosquito cell line following baculovirus infection [15]. To assess the effect on cell division in *Anopheles* Ag55 cells, we used the CPD eFluor 450 dye, which binds to cellular proteins and is evenly diluted with each division, enabling us to track proliferation by the decrease in fluorescence over time. Ag55 cells were infected with the *Yob*-expressing virus for 2 h, 4 h, 6 h, 8 h, or 24 h and stained the following day with CPD; cells were incubated with virus-free medium for an additional 24 h before harvesting them for analysis by flow cytometry (Additional file 4: Fig. S1A; right panel). In infected (EGFP+) cells, the CPD 450 intensity was comparable to that of the control cells stained immediately prior to flow cytometry analysis indicating a proliferative arrest (Additional file 4: Fig. S1F). In contrast, CPD 450 intensity was lower in the EGFP- population, implying that cells that were not infected with the baculovirus continued to undergo cell divisions. Of note, under the 24 h-virus exposure condition, both the EGFP- and EGFP+ cells showed a proliferative arrest, perhaps due to some viruses having already entered despite cells being still scored EGFP-, or a paracrine effect (Additional file 4: Fig. S1F).

### *Anopheles* host transcriptome responses upon baculovirus infection

**Fig. 3:**
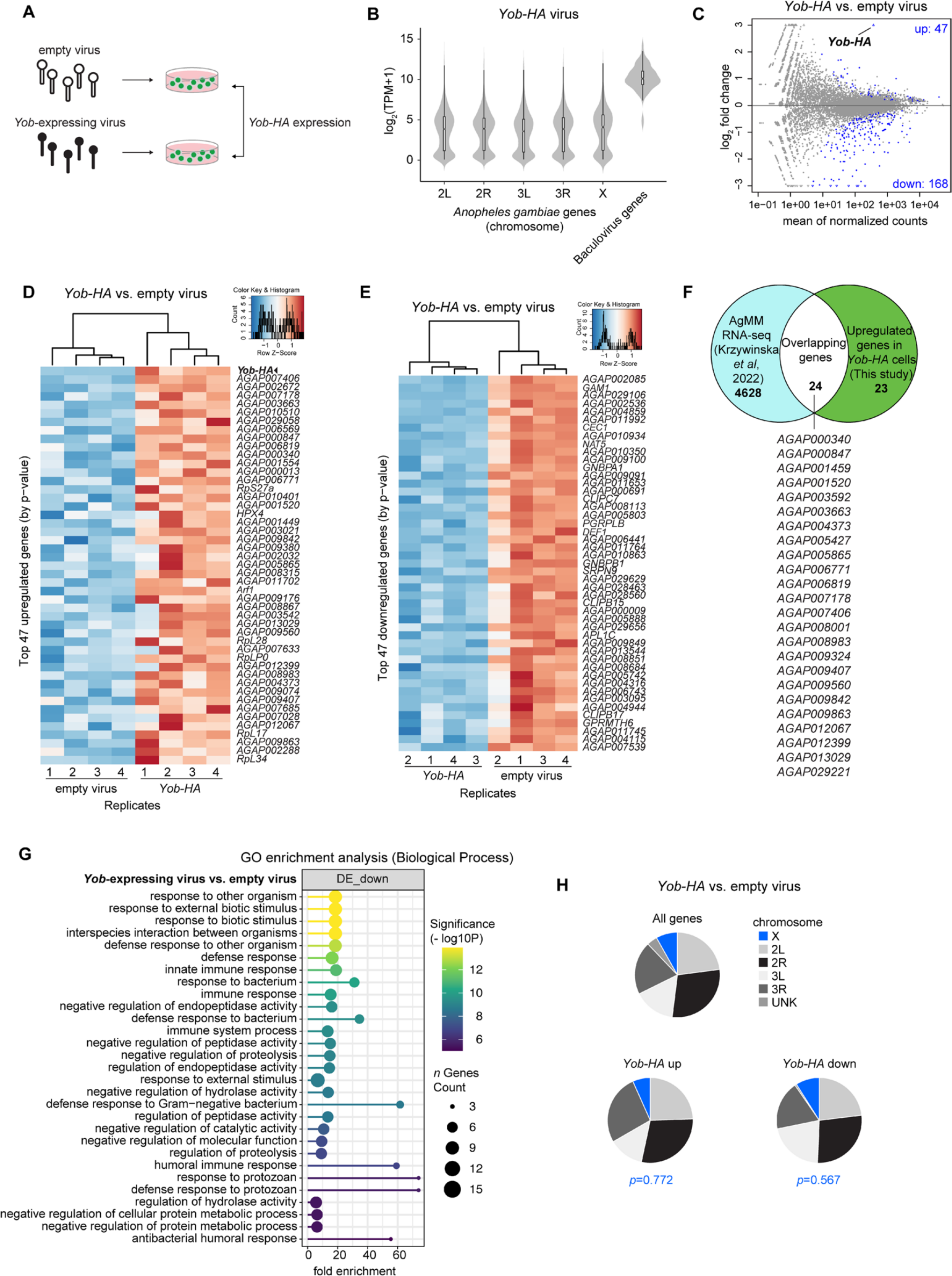
Gene expression changes upon *Yob* ectopic expression. **A** Scheme showing the experimental setup. **B** Violin plots of log_2_(FC) values of Ag55 *Yob*-expressing cells. The center line of the boxplot indicates the median. All genes with average read count > 0 were plotted. **C** MA plots from RNA-seq showing normalized read counts versus log_2_(FC) comparing infected *Yob*-expressing cells against the “empty virus” control. **D** Heatmap of all 47 upregulated genes in cells expressing *Yob*-*HA* compared with an “empty virus”. **E** Heatmap of downregulated genes in cells expressing *Yob*-*HA* compared with an “empty virus”; the top 47 genes by FDR are shown. **F** Venn diagram showing the overlap between the upregulated genes in **D** and the male-specific genes of the male AgMM cell line from [9]. **G** Gene Ontology (Biological Process) analysis of the differentially expressed genes from the RNA-seq of *Yob*-expressing cells versus cells infected with an “empty virus.” Downregulated gene sets (FDR<0.05) were analyzed independently and GO terms meeting a *p*_*adj*_ < 0.05 (Benjamini–Hochberg correction) were considered significantly enriched. The lollipop plot shows the fold enrichment of genes in the various classes, with the point size indicative of the gene count and color indicative of the *p*-value. The analyses were conducted with the GO Term tool on VectorBase. **H** Pie chart of the proportion of differentially expressed genes belonging to each mosquito chromosome comparing infected *Yob*-expressing cells against an “empty virus.” *P* values were obtained using a one-sided Fisher’s test for overrepresentation. *DE,* differentially expressed; *TPM,* transcripts per million; *UNK,* unknown

We next evaluated the effects of infection on the host and viral transcriptomes and conducted RNA sequencing (*n*=4 replicates of uninfected in comparison to Ag55 cells infected by the “empty virus” and the *Yob*-expressing baculovirus) (Figs. 2A and 3A). We mapped the data to a combined *Anopheles* and AcMNPV genome, including also the coding sequences (e.g., *EGFP*, *Blasticidin Resistance* gene) present in our constructs (Fig. 1A). Principal component analysis showed that the effects of virus infection far exceeded those induced by *Yob*-*HA* (Additional file 5: Fig. S2A). Almost all of the 156 baculoviral genes were expressed and displayed on average substantially higher expression levels compared with the host genes (Figs. 2B and 3B). Interestingly, although *Blasticidin-Resistance* (Additional file 5: Fig. S2B) and *EGFP* (Additional file 6: Table S4 and Table S5) RNA levels were similar between “empty virus” and *Yob*-expressing cells—indicating comparable infection rates—viral gene expression was higher in *Yob*-expressing cells (Figs. 2B and 3B and Additional file 5: Fig. S2C). To analyze the host transcriptome changes, we performed differential expression analysis with DESeq2 comparing cells infected with the “empty virus” control to uninfected control cells (no Baculovirus exposure). This uncovered 1551 upregulated and 1296 downregulated genes (FDR<0.05) (Fig. 2C). To identify the biological processes enriched among these differentially expressed genes, we performed Gene Ontology (GO) analyses. Our analyses revealed that, for example, cell cycle and DNA replication processes were downregulated in infected cells (Fig. 2D), consistent with CPD experiments showing an arrest in cell proliferation (Additional file 4: Fig. S1F). We were also interested in the chromosomal location of the differentially expressed genes and found that upregulated, but not downregulated, host genes upon infection with the “empty” baculovirus were overrepresented on the X chromosome (Fig. 2E). Using GO term analysis of all *Anopheles* X-linked genes showed that the X chromosome stands out in having a greater proportion of immune-responsive genes (Additional file 6: Tables S4 and S5), providing a possible explanation for the aforementioned overrepresentation. We also explored whether the infection-responsive genes are conserved and found that 90% have orthologs in *Aedes* mosquitoes, suggesting that this transcriptional and cellular response to baculovirus infection might be broadly conserved across insect cells.

### *Yob* ectopic expression upregulates enzymatic and metabolic genes in female mosquito cells

To explore the molecular roles of the maleness factor *Yob*-*HA* we next compared the transcriptome of *Yob*-expressing cells with the “empty virus”-infected control cells (Fig. 3A; DESeq2 FDR<0.05). As intended, *Yob* was robustly expressed in the *Yob-HA* condition (400 TPM), while no *Yob* transcripts were detected in control cells, consistent with the absence of a Y chromosome/the gene *Yob* in female Ag55 cells (Additional file 5: Fig. S2D). In addition, *Yob* expression induced 47 upregulated genes and 168 downregulated genes (Fig. 2C–E). These numbers are consistent with our transcriptome characterization of embryogenesis in *Anopheles*, where a total of 111 sex-biased genes were identified [32]. We compared our 47 upregulated genes to an expression dataset from the recently derived AgMM cells, the only known male *Anopheles* line [11]. The authors identified AgMM-specific genes by comparing the AgMM transcriptome against Sua5.1, an independently derived female line that has long been studied in the field [11]. We found that half of the upregulated genes in our dataset were among the AgMM-specific genes, including all of the RNA-metabolism genes (see below) (Fig. 2F). We also investigated the impact of *Yob*-*HA* expression on *dsx* splicing, which showed a decrease in the levels of the female-specific *dsxF* isoform, accompanied by an increase of the male-specific *dsxM* isoform (Additional file 5: Fig. S2E). Although the effects on *dsxM* were subtle, especially when compared with in vivo controls (male and female pupae, Additional file 5: Fig. S2E), they are consistent with previous data in female cells, where *Yob* mRNA transfection induces only 20–40% male-specific splicing [4].

We next investigated the functions of the genes differentially expressed by Yob-HA and performed GO analyses revealing significant GO terms such as “response to biotic stimulus” or “response to external stimulus” (Fig. 3G). No significant GO terms were obtained among the upregulated genes. Manual inspection of the upregulated gene group showed that those transcripts are mainly involved in enzymatic reactions (*AGAP006569*, acetyl-CoA synthetase; *AGAP001520*, protein phosphatase 1; *AGAP007028*, glucosyl/glucuronosyl transferases) as well as RNA and protein metabolism (*AGAP007406*, elongation factor 1-alpha; *AGAP005427*, RpL2; *AGAP009863*, eukaryotic translation initiation factor 4A; *AGAP003663*, ATP-dependent RNA helicase DBP2). Particularly notable was *AGAP000847*, whose *Drosophila* ortholog, Darkener of apricot (DOA), is directly involved in somatic sex determination in fruit flies [33]. DOA encodes a Cdc2-like kinase that phosphorylates components of the sex determination pathway, resulting in sex-specific splicing of *dsx* [34]. The upregulation of its *Anopheles* ortholog upon *Yob* expression makes it an intriguing candidate for a conserved role in mosquitoes. Together, these results suggest that *Yob*-*HA* induces a sex-specific transcriptome that shapes RNA metabolism, with some downstream regulators broadly conserved across insects, generally in line with the hourglass model of sex determination [35].

Lastly, we also assessed the effect on X chromosome transcriptional output, as Yob has been suggested to be an upstream factor inducing the male-specific splicing of the dosage compensation factor SOA [4, 6, 9], which in turn binds and upregulates the single male X chromosome [5]. In our conditions, we could, however, not find an overrepresentation of X-linked transcripts by DESeq2 (Fig. 3B right panel, Fig. 3H and Additional file 5: Fig. S2F). This piece of evidence suggests that either Yob is not the sole determinant to SOA splicing and X upregulation, or that our expression conditions in cells are not sufficiently strong enough to provide full masculinization as observed in vivo upon mRNA injection in embryos.

### Yob localizes to the nucleus without detectable chromatin binding activity

**Fig. 4:**
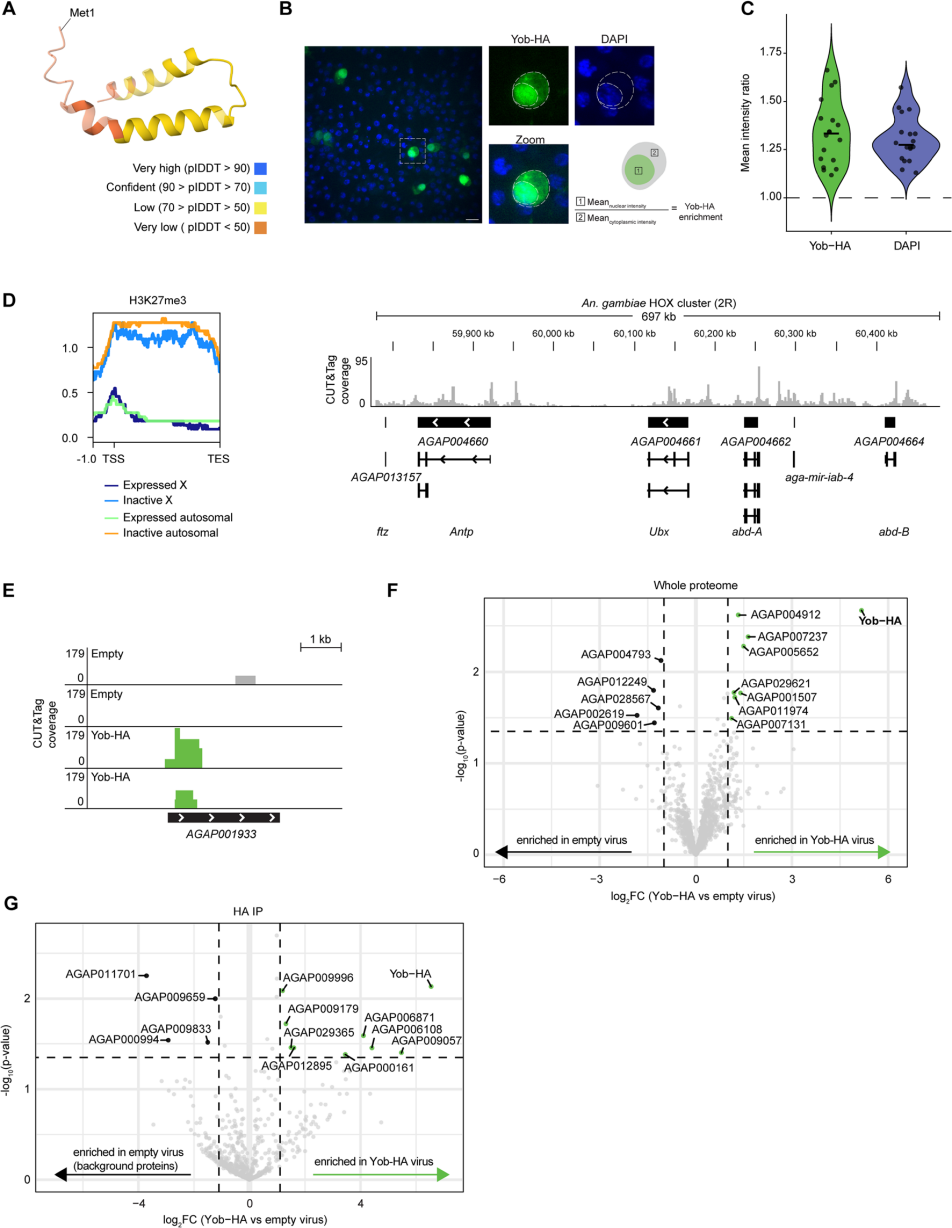
Yob is a nuclear protein that does not bind to chromatin. **A** Predicted secondary structure of Yob protein generated with AlphaFold. The model is color-coded by pIDDT confidence scores. The first amino acid is shown. **B** Representative microscopy pictures of endogenous fluorescence of EGFP in the Yob-HA construct in infected Ag55 cells. The right panel shows a magnification (zoom) of the area highlighted with a white dashed square. The pictures are a 3D view representation of *Z*-stacks; DAPI is shown in blue. The outer dashed circle represents the cell membrane and inner circle the nucleus. Scale bar = 10 µm. The drawing represents the Yob-HA enrichment. **C** Quantification of **B**. The violin plots show the mean fluorescence intensity distribution. Each dot represents the average of at least three quantified EGFP + cells per field of view. The center line represents the median. Yob-HA is represented in green, DAPI in blue. **D** Left: Metaplot of H3K27me3 profiling by CUT&Tag on inactive (<1 TPM) versus expressed genes (≥1 TPM) distributed along the gene body. Right: Genome browser snapshots of H3K27me3 CUT&Tag coverage along the HOX cluster. **E** Genome browser snapshot of the CUT&Tag coverage of the only peak with significant binding (FDR<0.05 in DiffBind comparing *Yob-HA* to the “empty virus” control).** F** Whole-proteome mass spectrometry experiment represented as a volcano plot, with log_2_ fold change (log_2_FC>1) between cells infected with the *Yob*-expressing virus compared with the “empty virus” on the *x*-axis and −log_10_ (*p*-value<0.05) on the *y*-axis. Black dots represent proteins depleted upon *Yob* ectopic expression, and green dots represent proteins with higher abundance in cells infected with the *Yob*-expressing virus; proteins without changes are shown in grey. Dashed lines indicate significance and fold-change cutoffs. **G** Yob-HA IP experiment where Yob interaction partners are mapped in comparison to a background control (IP in cells infected with the “empty virus” to account for unspecific background binding of the antibody). IP was performed on whole cell extracts from Ag55 cells using HA antibody. Dashed lines indicate significance and fold-change cutoffs. *Met1,* methionine 1; *TPM,* transcripts per million; *TSS,* transcription start site

Yob is a 56-amino-acid protein with no predicted domains. We performed AlphaFold modeling [24, 25], revealing that its structure consists of two alpha helices separated by a linker (Fig. 4A). In western blots against the HA-epitope present in our constructs, we detected the protein at 35 kDa, which is consistent with the production of a Yob-HA-EGFP fusion protein (Additional file 7: Fig. S3A). In line with the functional characterization (Fig. 3), AlphaFold modeling revealed that the fused protein maintains its helical structure as the untagged protein (Fig. 4A and Additional file 7: Fig. S3B). We investigated the cellular localization of Yob-HA by microscopy (Fig. 4B). Quantification of mean fluorescence intensity of EGFP signal in the nucleus (identified by DAPI staining) versus the cytoplasm revealed that Yob-HA was enriched in the nucleus (Fig. 4C). We found no canonical nuclear localization signal sequence in Yob [36–38], suggesting that Yob (especially in its untagged form as 56-amino-acid polypeptide) likely enters the nucleus by passive diffusion.

Because in our RNA-seq data *Yob*-*HA* expression triggered the upregulation of genes involved in different metabolic processes (enzymes and RNA/protein; see above), we next investigated whether Yob-HA acts as a transcription factor that either activates the upregulated genes or represses the downregulated transcripts by directly associating with chromatin. We employed CUT&Tag, a chromatin profiling technique that uses an pA-Tn5 transposase protein to direct any antibody (in our case against the HA-epitope) for recognition of chromatin-bound proteins or modified histones [19]. As a negative control, we used the cells infected with the “empty virus.” As controls, we profiled at the same time a conserved histone modification (histone H3 lysine 27 trimethylation, H3K27me3) as well as HA-tagged SOA, which binds to the X chromosome (dataset analyzed and published in [5]). After removing sequencing adapters, mapping to the *Anopheles* genome, and peak calling, we compared the significantly and differentially bound peaks between the samples. H3K27me3 profiling showed that, as expected for a histone mark linked to transcriptional repression [39], this modification is strongly enriched over inactive genes on both the X chromosomes and the autosomes, including genes within the HOX cluster (Fig. 4D). This, together with the data published in [5], validates the quality of these experiments. Comparing the *Yob*-*HA* versus the “empty virus” profile with DiffBind revealed only one significantly enriched peak at the *AGAP001933* gene (Fig. 4E). Its ortholog in the fruit fly—*bällchen*—encodes for a serine/threonine kinase involved in phosphorylation of the C-terminal tail of histone H2A [40] and the BAF protein [41]. This gene was neither upregulated nor downregulated in the transcriptome (Fig. 3), suggesting that this single peak is likely false positive. Taken together, these CUT&Tag results indicate that, despite localizing to the nucleus, Yob does not function as a classical transcription activator or repressor through direct chromatin binding.

### Yob stabilizes and regulates RNA metabolism factors in the nucleus

Since we could not find evidence that Yob-HA functions as a transcription factor, we next investigated whether it acts by binding and regulating other nuclear proteins. To this end, Ag55 cells were infected with *Yob*-expressing or “empty control” virus, EGFP-positive cells were isolated by FACS, and their proteomes were compared by mass spectrometry-based proteomics. Five proteins were downregulated, out of which three are metabolic enzymes. Eight proteins became significantly stabilized upon *Yob*-*HA* expression and were linked to metabolism and RNA-related processes (Fig. 4F and Additional file 7: Fig. S3D), one of which was also upregulated at the RNA level (*AGAP007237*). This finding, together with their higher intensity and abundance in comparison to the “empty virus,” implies that the enriched proteins are rather stabilized at the protein level by the presence of Yob-HA. Among them, AGAP004912 and AGAP005652 were of particular interest. These two proteins are DDX5 RNA helicases generally involved in RNA metabolism [42], which were proposed to regulate *An. gambiae* transposons via small RNA pathways [43]. In addition, CG10077 is the ortholog of AGAP005652 in *D. melanogaster*. In primordial germ cells of the embryo, the *AGAP005652* transcript immunoprecipitates with the Sex-lethal protein (Sxl) [44], which promotes female sex determination [45], suggesting that AGAP005652 could be similarly involved in mosquito male development.

To explore this further, we performed immunoprecipitation followed by mass spectrometry to identify proteins directly interacting with Yob-HA (Additional file 7: Fig. S3C, E). We identified four proteins that were more abundant in the “empty virus” control, representing nonspecific background binding. In contrast, ten proteins were significantly (−log_10_(*p*-value<0.05) and log_2_FC>1) enriched in Yob-HA, with Yob-HA itself being prominently enriched (log_2_FC=6.5). The proteins that co-enrich with Yob-HA play roles in RNA homeostasis (Fig. 4G and Additional file 7: Fig. S3E). The *Drosophila* ortholog of AGAP009057 is a subunit of the CCR4-NOT complex, a poly(A)-specific ribonuclease [46], and the ortholog of AGAP006108 is a cold shock domain-containing protein, also involved in RNA metabolism [47]. In *Drosophila*, the CCR4–NOT complex and cold shock domain-containing proteins interact together to regulate mRNA deadenylation [48]. On the basis of these data, we propose that Yob might exert its effect not as a classical transcription factor, but by stabilizing and regulating nuclear proteins involved in RNA regulatory processes.

## Discussion

Because conventional rearing methods are costly and labor-intensive, cell cultures are a great alternative tool to study mosquito biology. Previously, the use of baculovirus as a gene delivery vehicle has been demonstrated in mosquito *Aedes* cells [15], and here, we expanded its use to *Anopheles* cells to study endogenous genes. We cloned different promoters, allowing the desired expression levels of the gene of interest to be tuned. A few-hours pulse of virus incubation is sufficient for high infection rates, accompanied by substantial cellular (e.g., proliferative arrest) and transcriptome alterations with more than 1000 host genes becoming misregulated. Indeed, Ag55 is inherently responsive to infections [49], and thus it is vital to account for general host responses elicited by the baculovirus (e.g., by including the “empty virus” control) when studying endogenous genes, especially when those are expected to be involved in X chromosome regulation or cell proliferation. Using our cellular expression system, we found that the *Anopheles* X chromosome contains a great number of immune-responsive genes that, upon baculovirus infection, became upregulated. As a result, one must use carefully chosen controls when studying X chromosome dynamics using baculovirus or any other viral approaches. Besides controlling viral titers and accounting for the immune response using an “empty virus” as we did here and in our previous work [5], additional methodology, such as transgenic mosquitoes [5], can validate findings. Although we have not explored the transcriptome signature in *Anopheles* cells other than Ag55, the hemocyte-like origin of Sua5.1 and its widespread use to study immune functions [11] suggests that it would show similar responses upon baculovirus infection. Given the extent of gene expression changes observed by us in cells, it may be important to expand such characterizations of infection effects to tissues, as baculoviruses are increasingly being explored as in vivo gene delivery tools, e.g., in honeybees [50].

The *Yob*-expressing baculovirus, unlike the “empty virus,” did not replicate in Ag55 cells, despite comparable expression levels of the normalizer genes (*Blasticidin Resistance*) present in our constructs. This observation points out that *Yob* expression may perturb cellular homeostasis in female cells, potentially leading to their negative selection relative to non-infected cells. The transcriptomic changes induced by *Yob* were modest, yet some upregulated genes are involved in RNA metabolism and protein synthesis. The observation that the mosquito ortholog of DOA, a protein involved in *Drosophila* sex determination [44], is differentially regulated makes it a novel and intriguing candidate for future studies exploring a putative role in mosquito sex-specific gene expression and splicing. Moreover, the effects of Yob on alternative splicing of *dsx* and X chromosome hyperactivation were moderate or undetectable, respectively. This is in line with previous observations in cells [4] as well as with stable transgenic lines in vivo [6] or transfected larvae [4], where only moderate molecular and phenotypic changes were elicited. For our experiments in cells, this is unlikely an effect of insufficient expression, as we used the strongest *Act5c* promoter and we could readily detect *Yob* in both transcriptome and proteome. One possibility is that developmental timing is critical. *Yob* is highly transcribed shortly after early zygotic genome activation (ZGA), reaching its peak 5 h later [4, 32]. Its expression may trigger stable, irreversible effects that propagate in later developmental stages. This is also consistent with observations where the injection of *Yob* mRNA into embryos resulted in complete penetrance of the masculinization phenotype [4]. Such a two-phase system operates, e.g., in mammals, where early embryonic expression of the lncRNA *Xist* initiates stable X-chromosome silencing—partly through compartmentalization [51]. Once established, this silenced state does not *a priori* require continued *Xist* expression and persists even after the initial trigger is removed [52]. It will be interesting to characterize the molecular features that make Yob particularly powerful in embryos.

We found that Yob protein localizes to the nucleus but does not stably associate with chromatin, as indicated by CUT&Tag analysis. While the Y-linked mammalian SRY protein that determines maleness functions as a transcription factor, other master sex determination genes encode diverse types of molecules—for instance, a lncRNA in the Argentine ant [53] and a Piwi-interacting RNA (piRNA) in the silkworm [54]. Although we cannot yet definitively determine its molecular function, Yob is known to function as a protein [4], while our data suggest that it does not act as a transcription factor, further supporting the idea that a variety of upstream molecular mechanisms can give rise to distinct male and female phenotypes. In our dataset, Yob neither stabilized, destabilized, nor interacted with factors previously reported to function in *An. gambiae* sex determination (*Femaleless* [8]) or dosage compensation (*SOA* [5]). However, we found that DDX RNA helicases become stabilized after *Yob* expression. DDX proteins have diverse roles in RNA metabolism [55] and have been associated with the RNA silencing pathway of transposable elements and repeats [43]. The X chromosome of mosquitoes is enriched in repeats and transposable elements. One possibility could be that Yob-induced RNA helicases dampen the effects of X-encoded transposon hypertranscription due to dosage compensation and thus safeguard genomic stability. The interaction between Yob and a subunit of the CCR4–NOT complex (AGAP009057) is also noteworthy, as it connects RNA deadenylation and degradation with the translation machinery [56], potentially linking Yob to posttranscriptional regulation of gene expression and proteome dynamics.

## Conclusions

Using baculovirus for gene delivery in female *Anopheles* cells, we show that ectopic expression of the male sex-determining gene *Yob* reshapes the transcriptome and proteome, particularly affecting RNA metabolism. Importantly, the baculovirus system offers a reliable approach to study mosquito gene function, enabling future studies on sex-specific traits, gene regulation, and disease transmission.

While our baculovirus delivery tool provides a versatile platform to interrogate mosquito gene function, the use of an in vitro model presents certain limitations. First, immortalized cell lines lack the complex, cell-type-specific environments found in vivo, which may mask certain effects of our protein of interest. Most notably, this system lacks the native developmental context required for Yob’s full molecular function. Because Yob naturally acts during ZGA to trigger potentially irreversible developmental cascades, its ectopic expression in the female Ag55 cell line likely misses this critical temporal window. This likely explains the moderate effects on male-specific *dsx* splicing and lack of X chromosome upregulation. Second, ectopic expression of *Yob* appears to impose a strong negative selection in female Ag55 cells. As a result, the infected cells recovered for downstream analysis might represent a subpopulation that is more tolerant to *Yob* expression, potentially dampening the observed molecular phenotypes. Finally, to completely understand the mechanism of action of Yob and validate its interaction partners, future studies need to include in vivo mosquito models.

## Supplementary Information

Additional file 1: Table S1. Plasmid sequences.

Additional file 2: Table S2. Primer sequences.

Additional file 3: Table S3 Antibodies.

Additional file 4: Figure S1. Effects of baculovirus infection in Ag55 cells. **A** Schematic illustration of the experimental setup. **B** Effect of timing infection, in terms of percentage, on the number of EGFP+ cells represented as a bubble plot (left) and on cell viability represented as bar plot (right). **C** Scheme representing the number of green cells as a function of baculovirus concentration. **D** Line plots of changes in baculovirus DNA levels over time in infected Ag55 cells. Baculovirus genes were normalized to endogenous mosquito genes in uninfected cells. **E** Representative microscopy pictures of SF9 infected with the supernatant of either uninfected Ag55 and SF9 cells or infected Ag55 and SF9 cells with the “empty” and *Yob*-expressing viruses for 24 h. Scale bar: 50 µm. **F** Representative flow cytometry histograms of the effect of baculovirus infection in cell division in Ag55 cells. CPD, cell proliferation dye.

Additional file 5: Figure S2. Gene expression changes in *Yob*-expressing cells. **A** Principal component analysis plot on the replicates and conditions of the RNA-seq experiments. **B** Blasticidin resistance gene expression from RNA-seq in either uninfected Ag55 cells or infected with an “empty” or *Yob*-expressing virus. **C** Heatmap of the expression of the baculoviral genes in cells infected with the “empty” or *Yob*-expressing virus. The top 50 genes are shown. **D** *Yob-HA *expression levels from RNA-seq in either uninfected Ag55 cells or infected with an “empty” or *Yob*-expressing virus. **E** RT-qPCR analysis of RNA levels of the indicated genes, including the sex-specific *dsx* isoforms (*dsxM* = male isoform, *dsxF* = female isoform) in cells infected with an “empty” or *Yob*-expressing virus. Sexed pupae were used as wild-type controls. Each dot represents one biological replicate. The error bars represent the standard error calculated from the replicate values. *RpL19* is shown as a control gene. *Rps4*, *Rp49*, *RpL19*, and actin were used as normalization genes. *TPM,* transcripts per million.

Additional file 6: Table S4. Raw read counts for the RNA-seq dataset. Table S5. GO Term of all X-linked genes. The analyses were conducted with the GO Term tool on VectorBase. Immune-related pathways are highlighted.

Additional file 7: Figure S3. Yob-HA-EGFP is translated as a fused protein. **A** Representative immunoblot of protein extracts from Ag55 cells infected with an “empty” or *Yob*-expressing virus. RNA Pol2 is used as a loading control. **B** Predicted secondary structure of Yob-HA-EGFP protein generated with AlphaFold. Wild-type Yob (untagged) is shown in grey overlay. The Yob-HA-EGFP model is color-coded by pIDDT confidence scores. The first amino acid (Met) is indicated. **C** Cropped immunoblot of HA antibody IP with corresponding input samples. Whole protein extracts from Ag55 cells expressing Yob-HA or “empty virus” were used. RNA Pol2 is used as a loading control. **D** Bubble plots representing the results of mass spectrometry of the whole proteome mass spectrometry experiment of *Yob*-expressing cells. All the significant upregulated and downregulated proteins in each condition are shown. The color of the bubbles represents the measured intensity, and their size the number of unique detected peptides. **E** Bubble plots as in **D** from the Yob-HA IP-mass spectrometry experiment of cells compared to the IP samples of cells infected with the “empty virus” (representing unspecific background binding of the antibody). Names of nuclear proteins in **E** and **D** are labeled in blue. CNOT1 is both present in the nucleus as well as in the cytoplasm. ABCF2, ATP-binding cassette transporter family F member 2; AP2M1,AP-2 complex subunit mu-1; AP3D1, adaptor-related protein complex 3, delta 1 subunit; CNOT1, CCR4-NOT transcription complex, subunit 1; CSP, cold shock domain-containing protein; DDX5, DEAD-box helicase 5; dsx, doublesex; GPCR, G-protein coupled receptor; Met1, methionine 1; NOL6, nucleolar protein 6; OAT, ornithine--oxo-acid transaminase; PPIL3, peptidyl-prolyl cis-trans isomerase-like 3; PRCP, lysosomal Pro-X carboxypeptidase; RBM26, RNA-binding protein 26; RNA Pol2, RNA Polymerase 2; RpL49, ribosomal protein L49; RpS4, 40S ribosomal protein S4; SCRC1, Class C Scavenger Receptor; TPR-containing protein, tetratricopeptide repeat-containing protein; VDAC2, voltage-dependent anion-selective channel protein 2.

## Data Availability

The mass spectrometry proteomics data have been deposited to the ProteomeXchange Consortium via the PRIDE [57] partner repository with the dataset identifier PXD070102. The RNA-seq and CUT&Tag data have been deposited into the Gene Expression Omnibus database with the identifiers GSE310063 and GSE310064, respectively.
