# Supplementary material for: Effects of expressing a maleness gene in Anopheles gambiae cells using baculovirus as a gene delivery tool
Source: Parasit Vectors. 2026 May 7;19:266. doi: 10.1186/s13071-026-07411-3 (PMC13321433; doi:10.1186/s13071-026-07411-3)
Supplement: Supplementary file 2 — Additional file 2: Table S2. Primer sequences. [file 13071_2026_7411_MOESM2_ESM.pdf]

Application	ID	Sequence (5'-3')	Target	Ensembl-ID	Amplicon size (bp)
qPCR	q03	GCTGCCGCTGGTGATCTT	Rps4	*AGAP006871*	65
	q04	TCGTCACCTCGCTGTTGGT			
	q05	GCTATGATAAACTCGCTCCCAA	Rp49	*AGAP002122*	189
	q06	TCATCAGCACCTCCAGCTC			
	q07	AGCGTACCAAGGTTCGTGAG	RpL19	*AGAP004422*	100
	q08	GCGGTCTCCTCTTCCTTAGC			
	q09	GGTGTGGCGTCTCTGTTTCT	Actin	*AGAP001676*	85
	q010	TCGCTAGCTACTCCCGAGAT			
	q023	ATTCAAGCTCTTCCCCTACCG	Suppressor of cytokine signaling	*AGAP000880*	74
	q024	TCAGTACGCTCCGAAAGACAG			
	q025	CGCCATGTTGGTACGATGGAT	Epidermal retinal dehydrogen ase	*AGAP000275*	91
	q026	GATATCGGTGTGGACGGTGTC			
	q042	GACAACCACTACCTGAGCAC	EGFP	-	72
	q043	CAGGACCATGTGATCGCG			
	q044	TGAGGTCAAGACCACCTACA	mCherry	-	106
	q045	GATGGTGTAGTCCTCGTTGTG			
	q128	CAATCAGCCTGTGTACAAGTTC	Yob	*AGAP029221*	108
	q129	CTGATTGTTGTCCGTTCTGC			
	q267	ACAACGAAGCGTCGAAAAACA	ACNV annihilator	*Ac-35K/p35*	83
	q268	CTTTTCGGATTTGCCCCAGC			
	q269	ACAAATCCACAGGGCCTAGTTT	ACNV helicase	*Ac-helicase*	59
	q270	TGTCATCGAACCCACGATACC			
	rt3	AAAGCACACCAGCGGATCG	doublesex, male isoform	*AGAP004050*	129
	rt4	CACCGAGATGTTCTCGTCC			
	rt7	ACCATCGTTCAACCAATACC	doublesex, female isoform		114
	rt8	GATTGATTGATTGTAGAGTGG			
