# Supplementary material for: Effects of expressing a maleness gene in Anopheles gambiae cells using baculovirus as a gene delivery tool
Source: Parasit Vectors. 2026 May 7;19:266. doi: 10.1186/s13071-026-07411-3 (PMC13321433; doi:10.1186/s13071-026-07411-3)
Supplement: Supplementary file 3 — Additional file 3: Table S3 Antibodies. [file 13071_2026_7411_MOESM3_ESM.pdf]

Antibody target	Species	Source	Cat. No.	Application	Dilution
H3K27me3	Rabbit	Active Motif	39055	CUT&Tag	1:100
HA	Mouse	BIOZOL	BLD-901502	CUT&Tag	1:100
				WB	1:1,000
RNA Pol2	Mouse	Active Motif	39097	WB	1:1,000
anti-rabbit IgG	Guinea pig	Sigma-Aldrich	SAB3700890	CUT&Tag	1:100
anti-mouse IgG	Rabbit	Abcam	ab6709	CUT&Tag	1:100
Secondary HRP-coupled anti-mouse IgG (H+L)	Goat	Avantor	ROCK610-1319-0100	WB	1:2,000
