# Supplementary figures and images for: Effects of expressing a maleness gene in Anopheles gambiae cells using baculovirus as a gene delivery tool
Source: Parasit Vectors. 2026 May 7;19:266. doi: 10.1186/s13071-026-07411-3 (PMC13321433; doi:10.1186/s13071-026-07411-3)

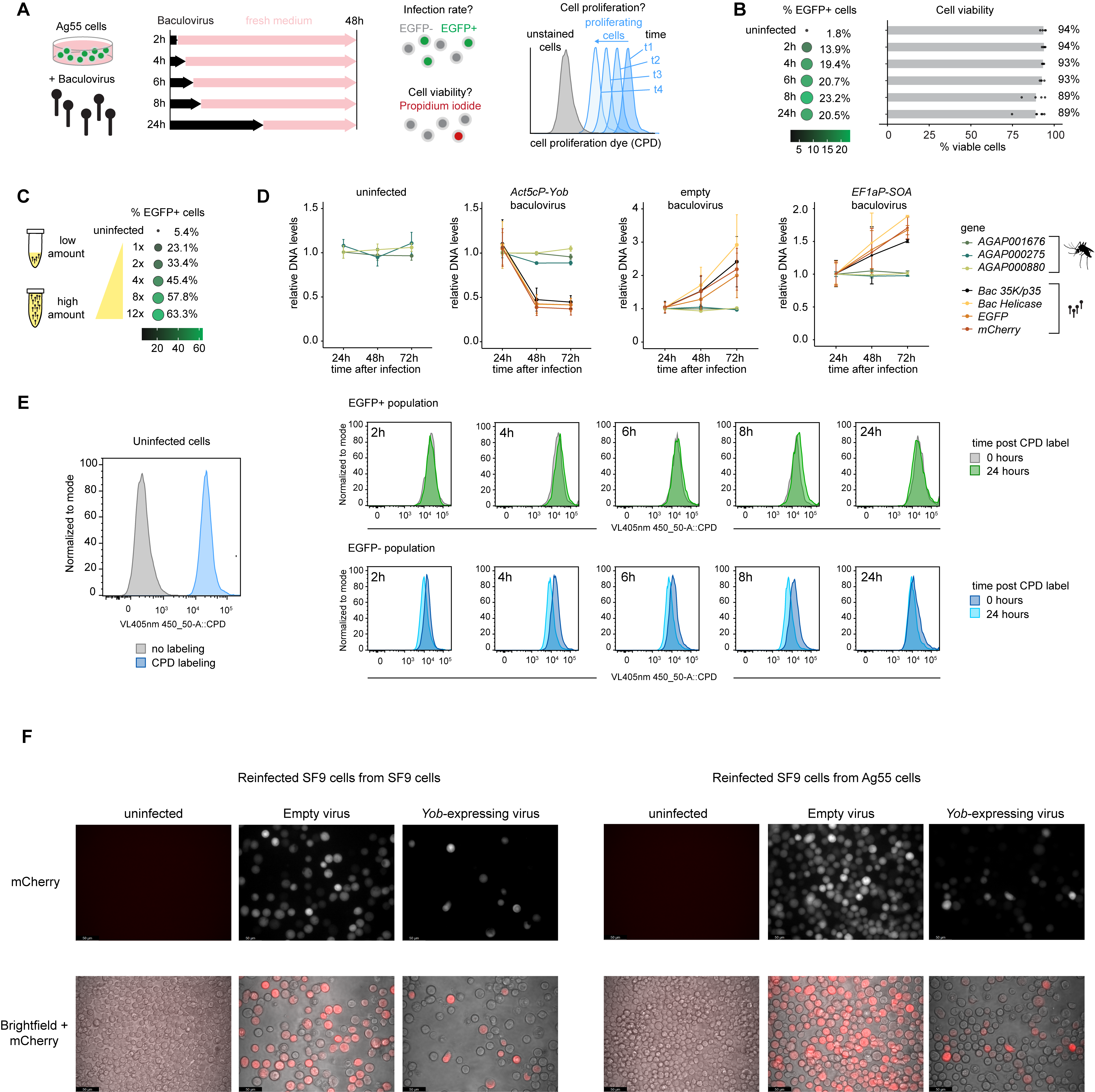

Supplement: Supplementary file 4 — Additional file 4: Figure S1. Effects of baculovirus infection in Ag55 cells. A Schematic illustration of the experimental setup. B Effect of timing infection, in terms of percentage, on the number of EGFP+ cells represented as a bubble plot (left) and on cell viability represented as bar plot (right). C Scheme representing the number of green cells as a function of baculovirus concentration. D Line plots of changes in baculovirus DNA levels over time in infected Ag55 cells. Baculovirus genes were normalized to endogenous mosquito genes in uninfected cells. E Representative microscopy pictures of SF9 infected with the supernatant of either uninfected Ag55 and SF9 cells or infected Ag55 and SF9 cells with the “empty” and Yob-expressing viruses for 24 h. Scale bar: 50 µm. F Representative flow cytometry histograms of the effect of baculovirus infection in cell division in Ag55 cells. CPD, cell proliferation dye. [file 13071_2026_7411_MOESM4_ESM.tif]

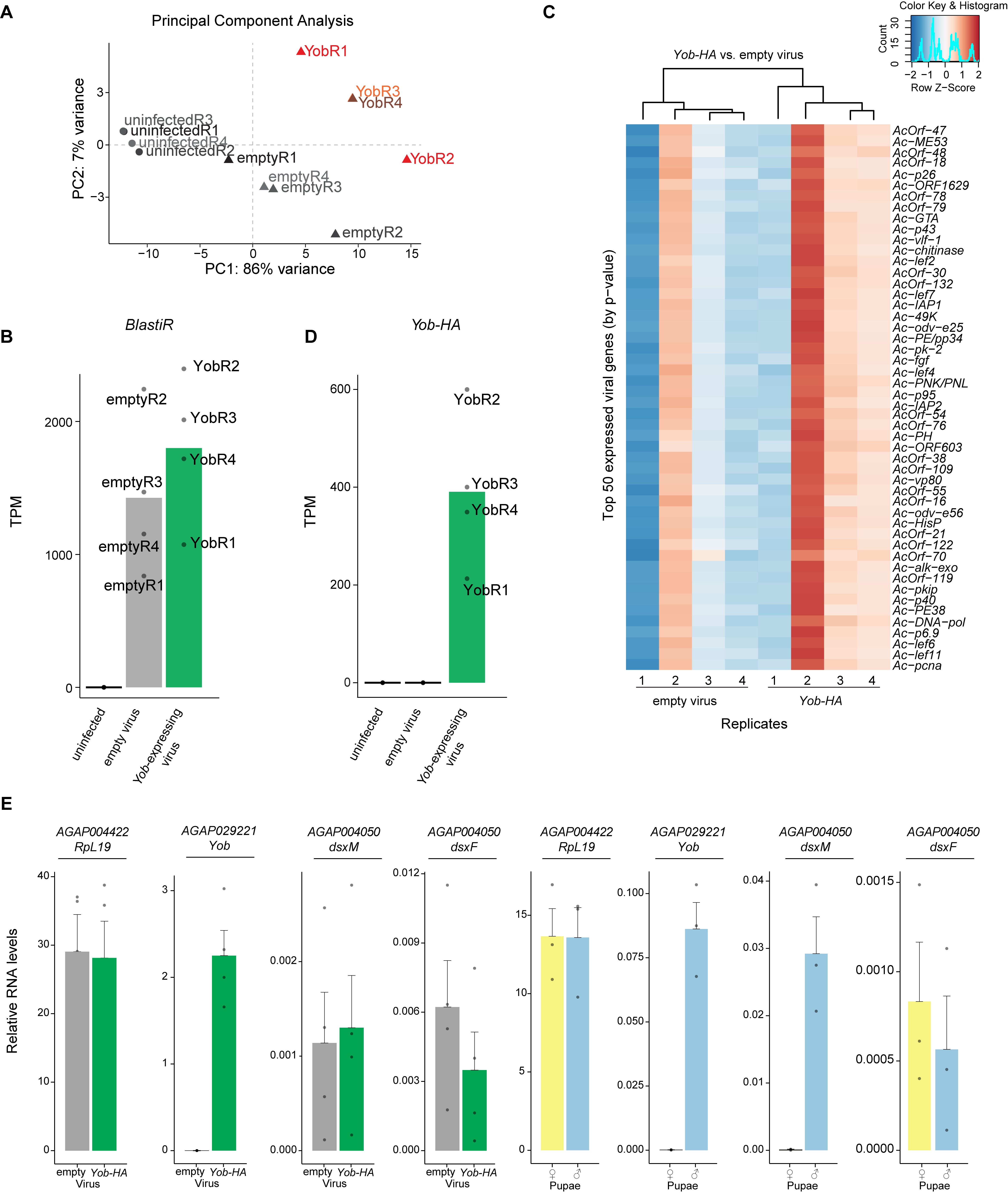

Supplement: Supplementary file 5 — Additional file 5: Figure S2. Gene expression changes in Yob-expressing cells. A Principal component analysis plot on the replicates and conditions of the RNA-seq experiments. B Blasticidin resistance gene expression from RNA-seq in either uninfected Ag55 cells or infected with an “empty” or Yob-expressing virus. C Heatmap of the expression of the baculoviral genes in cells infected with the “empty” or Yob-expressing virus. The top 50 genes are shown. D Yob-HA expression levels from RNA-seq in either uninfected Ag55 cells or infected with an “empty” or Yob-expressing virus. E RT-qPCR analysis of RNA levels of the indicated genes, including the sex-specific dsx isoforms (dsxM = male isoform, dsxF = female isoform) in cells infected with an “empty” or Yob-expressing virus. Sexed pupae were used as wild-type controls. Each dot represents one biological replicate. The error bars represent the standard error calculated from the replicate values. RpL19 is shown as a control gene. Rps4, Rp49, RpL19, and actin were used as normalization genes. TPM, transcripts per million. [file 13071_2026_7411_MOESM5_ESM.tif]

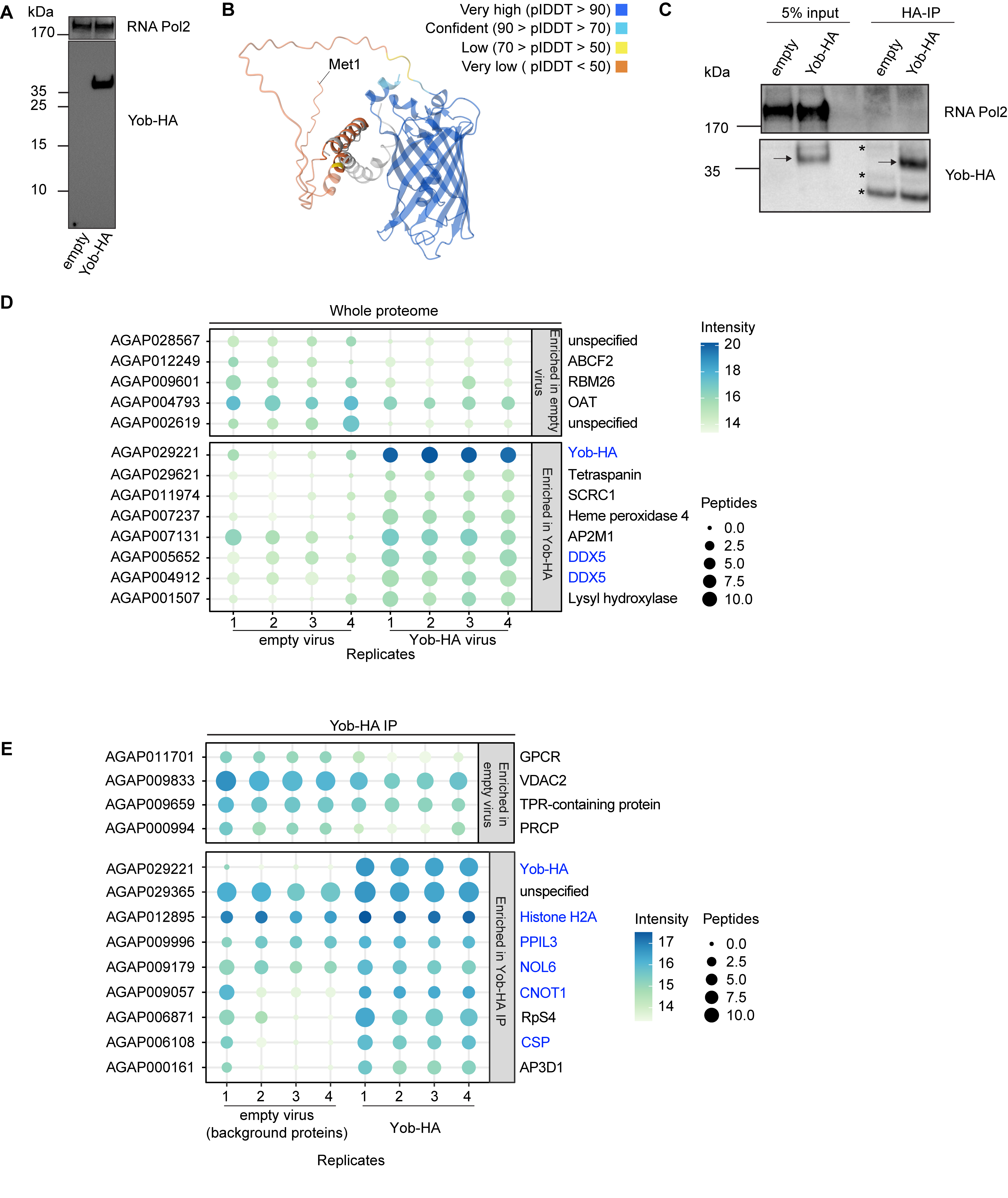

Supplement: Supplementary file 7 — Additional file 7: Figure S3. Yob-HA-EGFP is translated as a fused protein. A Representative immunoblot of protein extracts from Ag55 cells infected with an “empty” or Yob-expressing virus. RNA Pol2 is used as a loading control. B Predicted secondary structure of Yob-HA-EGFP protein generated with AlphaFold. Wild-type Yob (untagged) is shown in grey overlay. The Yob-HA-EGFP model is color-coded by pIDDT confidence scores. The first amino acid (Met) is indicated. C Cropped immunoblot of HA antibody IP with corresponding input samples. Whole protein extracts from Ag55 cells expressing Yob-HA or “empty virus” were used. RNA Pol2 is used as a loading control. D Bubble plots representing the results of mass spectrometry of the whole proteome mass spectrometry experiment of Yob-expressing cells. All the significant upregulated and downregulated proteins in each condition are shown. The color of the bubbles represents the measured intensity, and their size the number of unique detected peptides. E Bubble plots as in D from the Yob-HA IP-mass spectrometry experiment of cells compared to the IP samples of cells infected with the “empty virus” (representing unspecific background binding of the antibody). Names of nuclear proteins in E and D are labeled in blue. CNOT1 is both present in the nucleus as well as in the cytoplasm. ABCF2, ATP-binding cassette transporter family F member 2; AP2M1,AP-2 complex subunit mu-1; AP3D1, adaptor-related protein complex 3, delta 1 subunit; CNOT1, CCR4-NOT transcription complex, subunit 1; CSP, cold shock domain-containing protein; DDX5, DEAD-box helicase 5; dsx, doublesex; GPCR, G-protein coupled receptor; Met1, methionine 1; NOL6, nucleolar protein 6; OAT, ornithine--oxo-acid transaminase; PPIL3, peptidyl-prolyl cis-trans isomerase-like 3; PRCP, lysosomal Pro-X carboxypeptidase; RBM26, RNA-binding protein 26; RNA Pol2, RNA Polymerase 2; RpL49, ribosomal protein L49; RpS4, 40S ribosomal protein S4; SCRC1, Class C Scavenger Receptor; TPR-cont [file 13071_2026_7411_MOESM7_ESM.tif]
